# Evaluation of two different vaccine platforms for immunization against melioidosis and glanders

**DOI:** 10.3389/fmicb.2022.965518

**Published:** 2022-08-17

**Authors:** Sergei S. Biryukov, Christopher K. Cote, Christopher P. Klimko, Jennifer L. Dankmeyer, Nathaniel O. Rill, Jennifer L. Shoe, Melissa Hunter, Zain Shamsuddin, Ivan Velez, Zander M. Hedrick, Raysa Rosario-Acevedo, Yuli Talyansky, Lindsey K. Schmidt, Caitlyn E. Orne, David P. Fetterer, Mary N. Burtnick, Paul J. Brett, Susan L. Welkos, David DeShazer

**Affiliations:** ^1^Bacteriology Division, United States Army Medical Research Institute of Infectious Diseases (USAMRIID), Fort Detrick, Frederick, MD, United States; ^2^Department of Microbiology and Immunology, University of Nevada, Reno School of Medicine, Reno, NV, United States; ^3^Biostatistics Division, United States Army Medical Research Institute of Infectious Diseases (USAMRIID), Fort Detrick, Frederick, MD, United States; ^4^Department of Microbiology and Immunology, Faculty of Tropical Medicine, Mahidol University, Bangkok, Thailand

**Keywords:** *Burkholderia pseudomallei*, *Burkholderia mallei*, vaccine, mice, immune response, cellular immunity, humoral immunity, aerosol

## Abstract

*Burkholderia pseudomallei* and the closely related species, *Burkholderia mallei*, produce similar multifaceted diseases which range from rapidly fatal to protracted and chronic, and are a major cause of mortality in endemic regions. Besides causing natural infections, both microbes are Tier 1 potential biothreat agents. Antibiotic treatment is prolonged with variable results, hence effective vaccines are urgently needed. The purpose of our studies was to compare candidate vaccines that target both melioidosis and glanders to identify the most efficacious one(s) and define residual requirements for their transition to the non-human primate aerosol model. Studies were conducted in the C57BL/6 mouse model to evaluate the humoral and cell-mediated immune response and protective efficacy of three *Burkholderia* vaccine candidates against lethal aerosol challenges with *B. pseudomallei* K96243, *B. pseudomallei* MSHR5855, and *B. mallei* FMH. The recombinant vaccines generated significant immune responses to the vaccine antigens, and the live attenuated vaccine generated a greater immune response to OPS and the whole bacterial cells. Regardless of the candidate vaccine evaluated, the protection of mice was associated with a dampened cytokine response within the lungs after exposure to aerosolized bacteria. Despite being delivered by two different platforms and generating distinct immune responses, two experimental vaccines, a capsule conjugate + Hcp1 subunit vaccine and the live *B. pseudomallei* 668 Δ*ilvI* strain, provided significant protection and were down-selected for further investigation and advanced development.

## Introduction

*Burkholderia pseudomallei* (*Bp*) is a gram-negative pathogen and agent of melioidosis, a disease with forms ranging from acute and rapidly fatal to protracted and chronic. It is a major cause of sepsis and mortality in endemic tropical regions. The closely related species, *Burkholderia mallei* (*Bm*), is an obligate pathogen of mammals, including solipeds, and humans and causes the disease glanders (Galyov et al., 2010; Wiersinga et al., 2012; Currie, 2015; Titball et al., 2017; Dance and Limmathurotsakul, 2018). Besides causing natural infections, both organisms are potential biothreat agents; thus, they are both military and public health concerns (Cheng et al., 2005). *Bp* is especially worrisome due to its widespread distribution, its high infectivity (notably via inhalation), the complicated and prolonged treatment required, and its inherent resistance to antimicrobials (Galyov et al., 2010; Wiersinga et al., 2012; Dance and Limmathurotsakul, 2018). No approved vaccines currently exist for melioidosis or glanders.

The expanded understanding of the global distribution of *Bp* and its biothreat potential heighten the urgency to develop a vaccine that can blunt the public health impact of disease and prevent deliberate adversarial use of the pathogen (Cheng et al., 2005; Wiersinga et al., 2012; Limmathurotsakul et al., 2015, 2016; Titball et al., 2017; Dance and Limmathurotsakul, 2018). In addition to the factors described above, the major hurdles in developing an optimal prophylactic are the genetic and phenotypic plasticity of *Bp* strains and their ability to survive within host cells and evade host immune responses (Stevens et al., 2002; Galyov et al., 2010; Burtnick et al., 2011; Hayden et al., 2012; Dance and Limmathurotsakul, 2018). Furthermore, these attributes have likely contributed to the inability of vaccines to consistently confer sterilizing immunity after infection (Peacock et al., 2012; Limmathurotsakul et al., 2015; Titball et al., 2017; Burtnick et al., 2018).

Vaccine candidates have spanned a wide range of forms, including purified antigens (protein, carbohydrates) alone or combined with nanoparticles, inactivated whole cells, isolated outer membrane vesicles (OMV), live attenuated vaccine strains (LAV), and nucleic acids. Furthermore, their preparation has involved different formulations and supplemental immune-potentiators (Silva and Dow, 2013; Baker et al., 2017, 2021; Johnson and Ainslie, 2017; Khakhum et al., 2020, 2021; Wang et al., 2020). These are exemplified by OMVs prepared from a purine-deficient strain of *Bp* (Nieves et al., 2011; Baker et al., 2017), gold-linked nanoparticle-protein-LPS vaccine constructs (Tapia et al., 2021), LAVs deficient in essential amino acids and/or iron acquisition (Hatcher et al., 2016; Amemiya et al., 2019; Khakhum et al., 2021), and defined subunit vaccines that include OmpW (Casey et al., 2016) and the *Burkholderia* 6-deoxyheptan capsular polysaccharide (CPS) combined with various recombinant *Burkholderia* proteins (Burtnick et al., 2018).

Two experimental vaccine candidates, a LAV and a multi-component subunit vaccine, were evaluated in this investigation. Previous work characterized attenuated auxotrophic mutants of *Bp* (strain MSHR668) with deletions in genes required for the synthesis of the amino acid histidine or the branched chain amino acids (isoleucine, leucine, and valine). These strains were safe, even in immune-compromised NOD/SCID mice, yet still highly protective in the sensitive BALB/c mouse model of melioidosis (Atkins et al., 2002; Amemiya et al., 2019). They conferred complete protection against high-dose parenteral (intraperitoneal) challenge in the acute phase (3 weeks) and significant long-term survival in the chronic phase (60 days), as well as sterilizing immunity in most animals. Burtnick *et. al*. developed a defined vaccine consisting of *Burkholderia* CPS conjugated to the protein carrier Cross-Reacting Material 197 (CRM197) plus hemolysin co-regulated protein 1 (Hcp1) (Burtnick et al., 2012a, 2018; Titball et al., 2017). Hcp1, a virulence-associated type VI secretion system protein, is highly immunogenic and is serodiagnostic for melioidosis in human patients (Burtnick et al., 2011; Lim et al., 2015; Pumpuang et al., 2017; Phokrai et al., 2018). The CPS is a surface polysaccharide and essential virulence factor of *Bm* and *Bp* that has been used in diagnosis and vaccine-mediated protection (Parthasarathy et al., 2006; Burtnick et al., 2012a; Houghton et al., 2014; Marchetti et al., 2015). Immunization with the Conjugate + Hcp1 subunit vaccine was shown to elicit high antibody titers to the two antigens, strong T cell responses, and 100% survival for at least 35 days against an inhalational challenge with virulent *Bp*; significant though incomplete clearance of the bacterial burden was also achieved (Burtnick et al., 2018). In the current study, we also assessed the ability of AhpC (alkyl hydroperoxide reductase C) to augment the protection associated with Conjugate + Hcp1 immunization. AhpC detoxifies peroxides and is important in defending against peroxide-induced oxidative stress (O'Riordan et al., 2012; Reynolds et al., 2015; Guo et al., 2017). Enhanced T-cell immunity to AhpC correlates with survival in melioidosis patients (Dunachie et al., 2017) and it also functions as a protective immunogen against infection with various pathogens (Felgner et al., 2009; O'Riordan et al., 2012; Reynolds et al., 2015; Yi et al., 2016; Guo et al., 2017; Testamenti et al., 2020).

The objectives of the study described herein were to perform a direct comparison of two thoroughly developed and characterized vaccine prototypes, the LAV *Bp* strain MSHR668 harboring a deletion of an enzyme that is necessary for branched chain amino acid biosynthesis (668 Δ*ilvI*) and the defined Conjugate + Hcp1 subunit vaccine.

## Materials and methods

### Vaccine candidates and bacteria

The subunit vaccines were prepared by investigators at the University of Nevada, Reno. The candidates evaluated included CPS-CRM197 (Conjugate) plus recombinant Hcp1 lacking a His-tag, with adjuvants Alhydrogel and immunostimulatory CpG 2006 (ODN 7909) oligonucleotide (Conjugate + Hcp1) (Burtnick et al., 2018). This subunit vaccine was also tested in combination with an enzymatically inactive *Burkholderia* AhpC in which the Cys at position 57 was changed to a Gly (Conjugate + Hcp1 + AhpC), as described below (Schmidt et al., 2022). The LAV 668 Δ*ilvI* was constructed at the USAMRIID and derived from *Bp* strain MSHR668. The LAV was cultivated and prepared as described previously (Amemiya et al., 2019).

*Bp* strain K96243 is a commonly used virulent laboratory strain from Thailand and MSHR5855 is a virulent human clinical isolate from Australia (Van Zandt et al., 2012; Lim et al., 2015; Welkos et al., 2015; Bearss et al., 2017; Amemiya et al., 2019). For preparation of the challenge inoculum, a frozen stock aliquot of *Bp* K96243 was grown in 4% glycerol (Sigma Aldrich, St. Louis, MO) with 1% tryptone (Difco, Becton Dickinson, Sparks, MD) and 5% NaCl (Sigma Aldrich) broth (GTB) at 37°C with shaking at 200 rpm until late log phase, approximately 18 h (Trevino et al., 2018; Amemiya et al., 2019). The bacteria were harvested *via* centrifugation, resuspended in GTB, and quantified by OD_620_ estimation (Amemiya et al., 2019). The actual delivered dose of bacteria, as the number of colony forming units (CFU), was then verified by plate counts on sheep blood agar plates (Remel™, Thermo-Fisher Scientific, Waltham, MA). *Bm* strain FMH is a human clinical isolate derived from strain *Bm* ATCC 23344/China 7 (Srinivasan et al., 2001). *Bm* was grown on sheep blood agar plates or GTB; suspensions were prepared from broth cultures of GTB and quantitated for aerosol exposures as described for *Bp* (Bozue et al., 2016). M9 minimal medium as used to characterize maintenance of auxotrophic phenotypes.

### Animals and vaccination conditions

The animal research was conducted under an animal use protocol approved by the USAMRIID Institutional Animal Care and Use Committee (IACUC) in compliance with the Animal Welfare Act, PHS Policy, and other Federal statutes and regulations relating to animals and experiments involving animals. The facility where this research was conducted is accredited by the AAALAC International and adheres to principles stated in the Guide for the Care and Use of Laboratory Animals (National Research Council, 2011).

**Figure 1:**
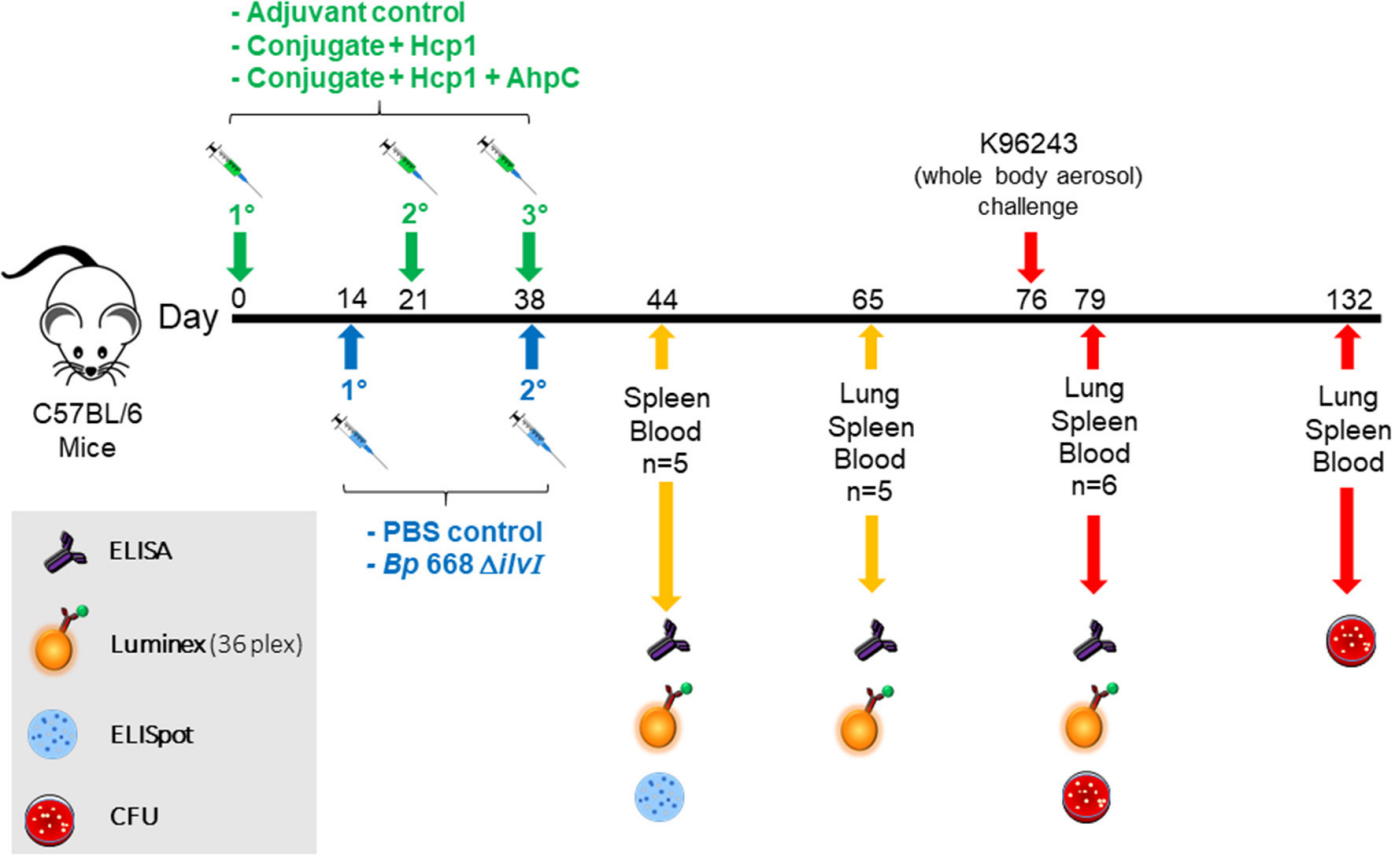
Overview of the immunization and challenge strategy for direct comparison of *Burkholderia* vaccine candidates that were challenged with *Bp* K96243. The numbers with the degree sign (°) denote vaccine prime and consecutive boost(s). The experiments that included mice challenged with *Bm* FMH or *Bp* MSHR5855 followed a similar timeline.

Immune-deficient NOD/SCID mice immune-deficient (Jackson Laboratories, Bar Harbor, ME) were used to demonstrate the extent of attenuation of the live vaccine strain (*Bp* 668 Δ*ilvI*) and were not used to evaluate adaptive immunity induced by the vaccine. C57BL/6 strain female mice were from Charles River (Frederick, MD) and were 7–10 weeks of age at time of vaccination (Figure 1). The vaccines were injected subcutaneously (SC) in a total volume of 200 μl, with the subunit vaccine given as 100 μl in each hind flank, as described below (Bearss et al., 2017; Burtnick et al., 2018; Amemiya et al., 2019). Control mice were inoculated with PBS alone or PBS with Alhydrogel and CpG (Adjuvant).

Mice given one of the subunit vaccine candidates, or adjuvant only, were vaccinated three times; the second and third doses of the subunit vaccines were administered 21 and 35–38 days after the first dose, respectively. The remaining groups of mice, given a LAV or PBS alone, were injected SC, in a total volume of 200 μl; the second dose was given 21–24 days after the first dose. Blood, spleen, and lung samples were collected from each vaccine and control group at various times before and after aerosol challenge.

### Exposure to aerosolized virulent challenge strains

Approximately 1 month after the last vaccine dose, the mice were exposed to aerosolized suspensions of *Bp* K96243, *Bp* MSHR5855, or *Bm* FMH, prepared as detailed above. For the exposures, mice were transferred to wire mesh cages and placed in a whole-body aerosol chamber within a class three biological safety cabinet located inside a BSL-3 laboratory. Mice were then exposed to aerosols of *Burkholderia* suspension created by a three-jet Collison nebulizer (Trevino et al., 2018). Samples were collected from the all-glass impinger (AGI) vessel and plated for CFU determinations to calculate the inhaled dose of *Bp* or *Bm*.

#### Clinical observations and sample collections

Mice were observed at least daily for 60–70 days for clinical signs of illness. Early intervention endpoints were used during all studies and mice were euthanized when moribund, according to an endpoint score sheet. Animals were scored on a scale of 0–5: 0–2 = no significant clinical signs (e.g., slightly ruffled fur); 3–4 = significant clinical symptoms such as subdued behavior, hunched appearance, absence of grooming, hind limb issues of varying severity and/or pyogranulomatous swelling of varying severity (increased monitoring was warranted); 5 = distress. Those animals receiving a score of ≥ were euthanized. Surviving animals were euthanized at the study endpoint and necropsied for tissue collection.

### Bacteriology

The tissues collected from necropsied mice included lung, spleen, and blood or liver. They were weighed andhomogenized with disposable PRECISION™ homogenizers (Covidien, Dublin, Republic of Ireland); the CFU of the homogenate were determined on sheep blood agar plates. Undiluted homogenate and 10-fold dilutions in PBS (Dulbecco's phosphate buffered saline, without Ca++ or Mg++) were plated in duplicate to determine sterility. The limit of detection was ~10–100 CFU/ml blood (depending upon the experiment) or 5 CFU/organ. After CFU determinations, samples were radiation-inactivated, sterility checked, and stored at −80°C while awaiting immunological analyses.

### Immune response assays

#### ELISAs

Immunoglobulin G (IgG) titers in vaccinated mice were determined by ELISA as described previously (Trevino et al., 2018). The capture reagents included purified antigens [Hcp1, CPS, AhpC, and type A O-polysaccharide (OPS)] and whole-cell-derived antigens to include radiation-inactivated *Bp* K96243 cells (BpK) or *Bm* FMH cells (Bm FMH). The purified and whole-cell-derived antigens were plated at 2 and 10 μg/ml, respectively. The Hcp1, CPS, AhpC, and OPS antigens were purified essentially as described previously (Burtnick et al., 2012a,b, 2018; Reynolds et al., 2015; Dunachie et al., 2017; Schmidt et al., 2022). The antibody titer results are reported as the geometric mean (GM) and geometric standard error (GSE) of the reciprocal of the highest dilution resulting in a mean OD of at least 0.100 ± 1 SD at 450 nm with a reference filter (570 nm). The limit of detection was a reciprocal titer of 50 and samples with an antibody titer of <50 were considered negative. In some ELISAs, subclass IgG1 and IgG2c titers were also determined in addition to complete IgG, as described before (Martin et al., 1998). All of the labeled secondary antibodies used in the ELISAs were goat anti-mouse IgG (or IgG subclass) obtained from Southern Biotechnology Associates, Inc. (Birmingham, AL).

#### Cellular immunity

##### Splenocyte collection

Spleens were collected from necropsied C57BL/6 mice, and the splenocytes were isolated and prepared for *in vitro* analysis as described previously (Trevino et al., 2018; Amemiya et al., 2019; Cote et al., 2021). Splenocytes were diluted to a concentration of 10^7^/ml in RPMI complete medium (ThermoFisher, Grand Islands, NY) and 4 × 10^6^/ml in CTL-Medium (Cellular Technology Limited, Cleveland, OH) with 1% L-Glutamine for the Luminex and ELISpot *in vitro* stimulation assays, respectively. Antigens used in the re-stimulation assays included Hcp1 and AhpC; all were tested at 5 μg/ml.

##### ELISpot assays

The assay was performed as described by the manufacturer (ImmunoSpot, Cleveland, OH). Splenocytes were incubated with Hcp1 or AhpC (5 μg/well) to stimulate the cells. Specifically, cells from each mouse were tested in duplicate in each of four independent stimulation conditions. A solution of phorbol 12-myristate 13-acetate (PMA; 100 ng/mL) and ionomycin (0.5 μg/mL) was used as the positive control stimulant and resulted in uniformly strong signals, while medium only wells were used as a negative control. Spots were scanned and analyzed using an automated ELISpot reader (CTL-Immunospot S6 Analyzer, CTL,). The T-cell responses were assessed as spot-forming cells (SFC), adjusted to 10^6^ cells per well, which was automatically calculated by the ImmunoSpot^®^ software for each stimulation condition and the medium-only control.

##### Cytokines/chemokine analysis

Cytokine and chemokine levels were assayed in spleen and lung homogenates and in restimulated splenocytes, as previously described (Trevino et al., 2018). Prior to analysis, the samples were thawed, centrifuged at 1,000 × g for 10 min, and the supernatant was then examined for cytokine expression by Luminex Mag Pix 36-plex mouse panel (Thermo Fisher Scientific, Grand Island, NY, USA) as per manufacturer directions.

### Statistical analyses

#### Survival

Survival curves of the vaccinated and control mice were estimated with the Kaplan-Meier method and were compared statistically using the log-rank test with Graph Pad Prism 9.0 (San Diego, CA) and SAS version 9.4 (Cary, NC). Significant differences in survival rates at days 7, 21 and 60 or 70 after virulent challenge were determined using the Fisher Exact test. The time-to-morbidity (TTM) values were expressed as the median and interquartile range, and were compared with the log-rank test using SAS version 9.4.

#### Mouse tissue bacterial viable counts

The viable counts of *Bp* or *Bm* from organ and blood samples were compared using the Exact rank-sum test (two-sample Wilcoxon test). The data used were log-transformed CFU values, adjusting the log_10_ to 0 when no CFU were detected, and the results summarized as GM and geometric standard deviation (GSD) values.

#### Comparisons of antibody levels

The ELISA antibody titers, determined as described above, were log transformed and the GM and GSE data analyzed by Welch's *t*-test or the two-sample exact Wilcoxon test.

#### Comparisons of cytokine levels

Cytokine concentrations determined in Luminex assays were log transformed and the GM values compared by the Welch's *t*-test or an Exact rank-sum test (two-sample Wilcoxon test). The GM and GSE were reported. The Welch's *t*-test was also applied to log-transformed ELISpot values.

## Results

### Characterization of the *Bp* 668 *ΔilvI* vaccine candidate

#### Attenuation of *Bp* strain 668 Δ*ilvI* as evaluated in the immune-deficient NOD/SCID mice

The mutant strain 668 Δ*ilvI* is highly attenuated in the sensitive BALB/c mice as shown previously; doses of 10^6^ CFU given by the SC route were non-lethal but protected against high dose IP challenge with *Bp* strain K96243 (Amemiya et al., 2019). To more stringently evaluate the extent of attenuation, 668 Δ*ilvI* was examined in mice with a non-obese diabetic (NOD)/severe combined immunodeficiency (SCID) background. NOD/SCID mice are impaired in the development of T and B cells and have defective natural killer (NK) cells. We inoculated NOD/SCID mice (*n* = 10) IP with ~1.4 × 10^4^ CFU of 668 Δ*ilvI*, which was equivalent to 106 LD_50_ of the wild-type MSHR668 in BALB/c mice (Welkos et al., 2015; Amemiya et al., 2019). As a control, we inoculated another group of NOD/SCID mice with 8.6 × 10^3^ CFU (equivalent to 65 LD_50_) of wild-type MSHR668, and observed the mice for 21 days. All mice challenged with MSHR668 succumbed by day 10 post-infection. In contrast, all NOD/SCID mice inoculated with 668 Δ*ilvI* survived for the duration of the experiment, 21 days post-challenge (*p* < 0.0001). At the end of the study, the ten survivors were euthanized and their spleens, livers and blood were examined. The samples from 9 of 10 mice inoculated with 668 Δ*ilvI* were sterile. One of the infected mice harbored six CFU in the spleen on day 21, but otherwise appeared healthy at the time of euthanasia. The isolates were tested for growth on M9 medium and they retained the 668 Δ*ilvI* phenotype. These results demonstrate that 668 Δ*ilvI* is highly attenuated in immune defective mice.

#### Survival and dissemination of *Bp* 668 ΔilvI in C57BL/6 mice

The *in vivo* survival and dissemination of 668 Δ*ilvI* was also evaluated in C57BL/6 mice. Mice were vaccinated SC with approximately 1.06 × 10^7^ CFU of 668 Δ*ilvI* and on days 1, 2, 3, 4, 7, 10, and 15 post-vaccination, five mice per time point were euthanized. Spleens were homogenized and serial dilutions were spread onto sheep blood agar plates to determine strain viability. *Bp* strain 668 Δ*ilvI* persisted in the spleens of vaccinated animals for 4 days, but was cleared by day 7 (Supplementary Figure 1). In addition, 100 μl of blood from each vaccinated animal was spread onto agar plates to assess bacteremia. No bacteria were isolated from the blood of any of the animals on days 1, 2, 3, 4, 7, 10, and 15 post-vaccination. Twenty of the mice were given a booster dose (1.4 × 10^7^ CFU) of the 668 Δ*ilvI* vaccine strain on day 21 and the same procedure was followed on days 1, 2, 3 and 4 post-vaccination. The blood and spleens from five animals per day were examined and no CFU were found. This result suggests that 668 Δ*ilvI* persists for <7 days in the spleens of animals after the first vaccine dose and is rapidly cleared after the second dose of vaccine.

### Comparison of subunit and live vaccines for efficacy against *Bp* in a C57BL/6 mouse aerosol model of infection

We compared the efficacy of the live 668 Δ*ilvI* strain with two candidate subunit vaccines for melioidosis. The three experimental vaccines (and doses administered) were as follows: (1) the two component subunit vaccine candidate (Conjugate + Hcp1), with each dose having ~0.25 μg CPS as a conjugate, 0.5 μg Hcp1, 250 μg Alhydrogel, and 10 μg CpG 2006; (2) the two component subunit vaccine candidate plus 0.5 μg of AhpC (Conjugate + Hcp1 + AhpC); or (3) the live vaccine candidate *Bp* strain 668 Δ*ilvI* (~1.0 × 10^7^ CFU/mouse target dose); control groups received either PBS alone or Alhydrogel with CpG adjuvant (Adjuvant). Three doses of the experimental subunit vaccines and two doses of 668 Δ*ilvI* vaccine were given (Figure 1). The C57BL/6 mice were exposed to a challenge with aerosolized *Bp* K96243 approximately a month (38 days) after the last vaccination. The final inhaled challenge dose was approximately 1.35 × 10^3^ CFU, equivalent to 3.4 LD_50_. Tissue and blood were collected for immunoassays at several time points throughout the study (Figure 1).

#### Efficacy

**Figure 2:**
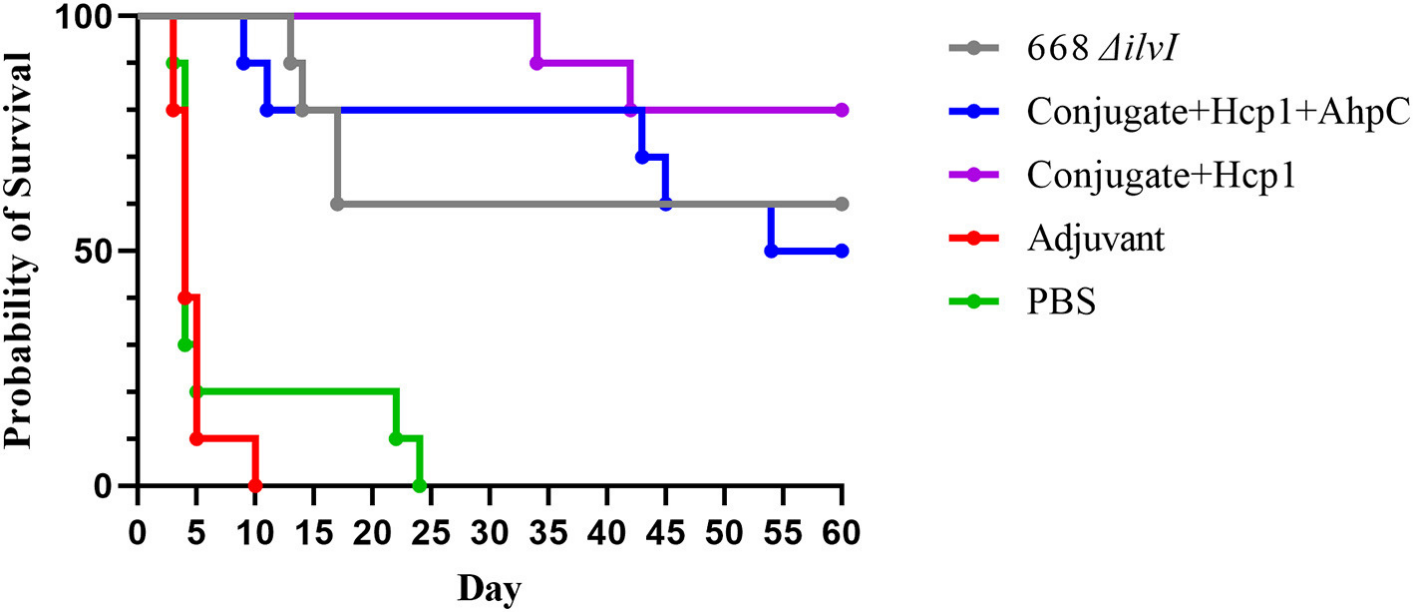
Survival curves of vaccinated C57BL/6 mice challenged with *Bp* K96243. Mice (*n* = 10/group) were exposed to 3.4 LD_50_ (1.35 × 10^3^ CFU) by the aerosol route and monitored for 60 days post challenge. The PBS control represented truly naïve mice and the adjuvant control confirmed that protection was not afforded to the mice due to non-specific immune stimulation associated with alhydrogel and CpG injections.

The mouse survival curves for the *Bp* K96243 challenge are shown in Figure 2. The Adjuvant control mice all succumbed or were euthanized by day ten post-challenge, and the PBS control mice all succumbed or were euthanized by day 24 post-challenge. At the end of the study, 50% of the Conjugate + Hcp1 + AhpC mice, 60% of the 668 Δ*ilvI* mice, and 80% of the Conjugate + Hcp1 mice had survived. The day 21 and 60 day survivorship of the Conjugate + Hcp1 mice was significantly greater than that of both control groups (*p* = 0.0007) but not of the Conjugate + Hcp1 + AhpC or 668 Δ*ilvI* mice. The mean TTM of the Conjugate + Hcp1 group non-survivors was also significantly greater (38.0 days) than that of both control groups (*p* < 0.0001) but not the Conjugate + Hcp1 + AhpC or 668 Δ*ilvI* groups (Figure 2, Supplementary Figure 2). Thus, the efficacies of the vaccinated mice groups could not be distinguished statistically. The majority of the control mice (Adjuvant or PBS) had succumbed to infection or were euthanized by day 5. These mice exhibited signs of acute disease (i.e., labored breathing and significant ruffling of fur). The vaccinated mice began to succumb or reach euthanasia criteria on day 9, with these few early deaths in the vaccinated mice showing similar but milder signs of acute disease compared to the control mice. The final phase of disease in this C57BL/6 mouse model began ~30 days after infection. These mice showed a different disease course which often included externally visible pyogranuloma formation and early signs of diminished mobility (i.e., altered gait or rear-leg issues generally leading to single and then double rear leg paralysis which triggered euthanasia intervention). These differences in clinical presentations underscore the complexity of melioidosis and the challenges with vaccine protection.

#### Bacteriology

**Figure 3:**
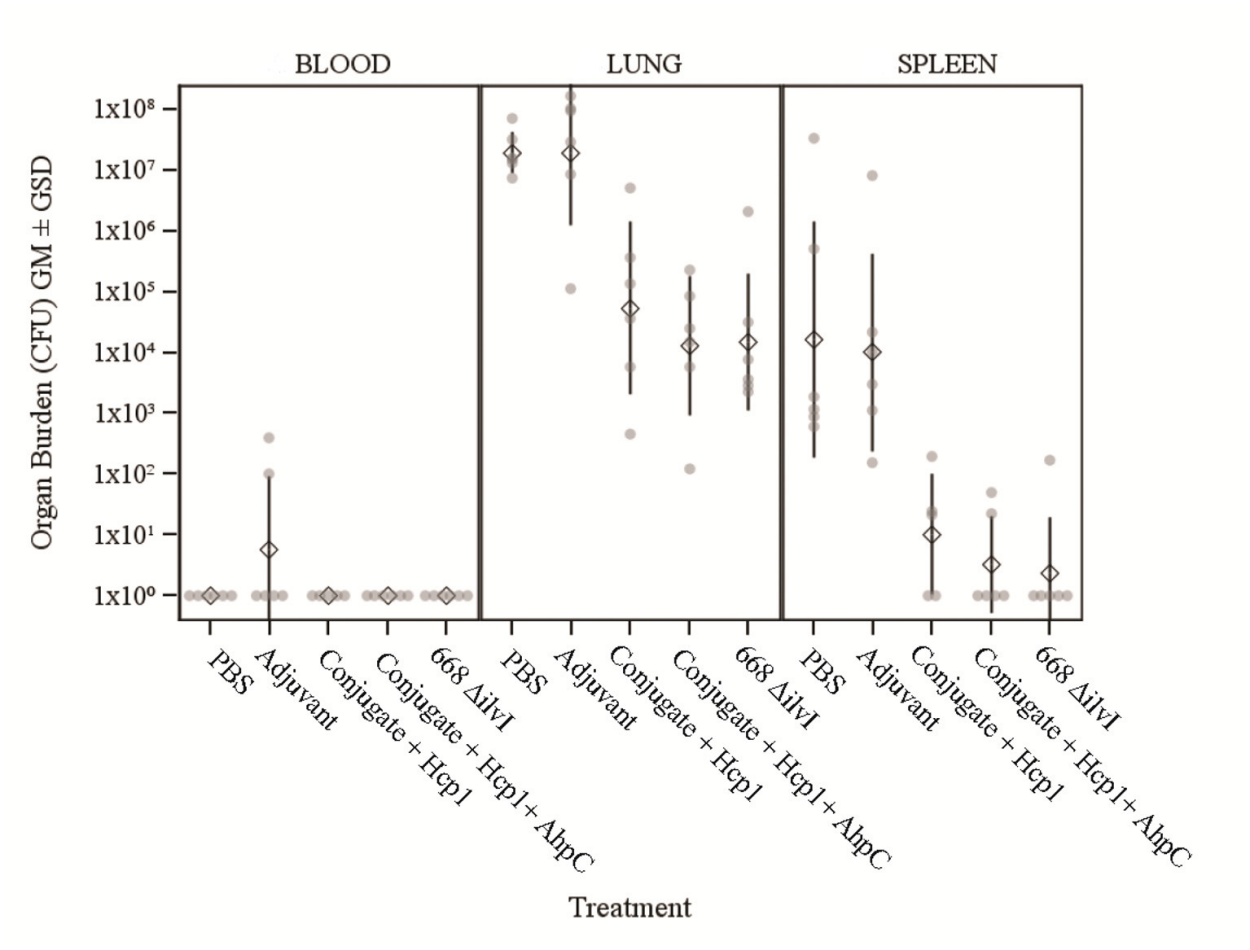
The recovery of bacteria as determined by CFU from blood, spleens and lungs of C57BL/6 mice 3 days post-challenge with *Bp* K96243. The samples were from six mice/group. The left axis represents CFU/ml (blood) or CFU/g (lung and spleen). The individual points represent one animal, and the baseline points indicate the remaining survivors with no detectable CFU. The diamond-shaped symbol and vertical line are the geometric mean and standard deviation, respectively. The limits of detection were 100 CFU/ml blood and 5 CFU/g spleen or lung homogenate.

The number of viable bacteria present in the blood, lungs, and spleens of six animals per group were determined on day 3 post-challenge. The only mice with detectable bacteria in the bloodstream were two Adjuvant control mice (1.00 and 4.00 × 10^2^ CFU/ml) as shown in Figure 3. The geometric mean (GM) concentration of bacteria in the lungs of the PBS and Adjuvant controls was of 2.00 × 10^7^ and 1.90 × 10^7^ CFU/g, respectively; and the concentrations were 100–1,000-fold lower (1.30–5.40 × 10^4^ CFU/g of lung tissue) in the vaccine groups (Figure 3), *p* = 0.015–0.002. The results suggest that by 3 days after challenge the vaccinated animals are able to clear or reduce the *Bp* infection quicker than the unvaccinated control animals. The spleens of the PBS and Adjuvant control animals had GMs of 1.64 × 10^4^ and 1.00 × 10^4^ CFU/g, respectively, there were at least three logs fewer bacteria in spleens of the three vaccine groups (GMs of 0.23–1.00 × 10^1^ CFU/g), all of which were significantly lower than the controls (*p* = 0.009–0.002), as shown in Figure 3. Splenic clearance was best in the 668 Δ*ilvI* and Conjugate + Hcp1 + AhpC vaccinated groups in which only one and two mice, respectively, had detectable bacteria. Thus, an increased rate of bacterial clearance in the blood, lungs and spleens at day 3 post-challenge was associated with the increased survival rate of the vaccinated animals as compared to the controls (Figure 2). The number of viable bacteria present in the blood, lungs and spleens of all surviving mice were also determined on day 60 post-challenge (Table 1). Three Conjugate + Hcp1 survivors had a high GM concentration (1.34 × 10^7^ CFU/g of lung) whereas lung samples from two Conjugate + Hcp1 + AhpC or 668 Δ*ilvI* vaccinated mice had approximately four logs less, *i.e*., GM concentrations of 1.14–2.51 × 10^2^/g.

**Table 1:** Detection of bacteria in mice surviving challenge with *B. pseudomallei*.

**Group**	**# Survivors^a^**	**# Positive samples**	**Organ (# mice)^b^**	**CFU/gram**	
				**GM^c^**	**GSD^c^**
Conjugate + Hcp1	8	3	Lungs (3)	1.34 × 10^7^	1.48
Conjugate + Hcp1 + AhpC	5	3	Lungs (2)	2.51 × 10^2^	4.76
			Spleen (1)	6.30 × 10^2^	NA^d^
668 *ΔilvI*	6	2	Lungs (2)	1.14 × 10^2^	1.39

a n = 10 mice/group before challenge. The data were from survivors on day 60 after challenge.

b The organ and number of mice from which had bacteria were detected.

c The geometric mean (GM) and geometric standard deviation (GSD).

d NA, not applicable.

#### Serum antibody responses

Antibody titers to six antigens were assessed by ELISA on sera collected at two time points before aerosol challenge. Table 2 shows the total IgG responses 6 days after the last vaccine dose (day 44). The mean anti-BpK titer induced by the LAV 668 Δ*ilvI* vaccine was significantly greater than those of the control groups (*p* = 0.0079); the 668 Δ*ilvI* anti-BpK titer was also 4.6× and 8.7× higher than the subunit (+/– AhpC, respectively)-vaccinated mice, but these were not significant. In contrast, the anti-Bm FMH titers of the two subunit groups were substantially higher than that of the 668 Δ*ilvI* mice, with *p* = 0.016 and 0.055, respectively for each subunit titer vs. 668 Δ*ilvI* titer. As expected, sera from the 668 Δ*ilvI* mice were more responsive to the OPS antigen. The two subunit vaccines induced the highest titers to the Hcp1 and CPS antigens compared to the two controls and 668 Δ*ilvI* (*p* = 0.0079); the differences in GM titers between these three latter groups were negligible and not significant. Also, the anti-Hcp1 titers of the Conjugate + Hcp1 group were more than 2.6-fold higher than those of the Conjugate + Hcp1 + AhpC vaccinated mice, although they were not significantly different. As expected, the only mice exhibiting a substantial antibody response to AhpC were those vaccinated with Conjugate + Hcp1 + AhpC, with a mean reciprocal dilution titer of 200,316 (*p* = 0.0079 for all comparisons) (Table 2).

**Table 2:** Total IgG response 6 days post-vaccination (day 44) in C57BL/6 mice.

**Antigen^a^**	**Groups^b^**	**IgG^c^**
BpK	PBS	79 (1.29)
	Adjuvant	57 (1.27)
	Conjugate + Hcp1	2,016 (2.25)
	Conjugate + Hcp1 + AhpC	3,850 (2.36)
	668 *ΔilvI*	17,549 (1.64)
Hcp1	PBS	174 (1.34)
	Adjuvant	83 (1.24)
	Conjugate + Hcp1	145,645 (1.60)
	Conjugate + Hcp1 + AhpC	55,452 (2.17)
	668 *ΔilvI*	126 (1.21)
CPS	PBS	50 (1.00)
	Adjuvant	50 (1.00)
	Conjugate + Hcp1	70,086 (2.18)
	Conjugate + Hcp1 + AhpC	64,101 (1.79)
	668 *ΔilvI*	60 (1.20)
AhpC	PBS	55 (1.12)
	Adjuvant	50 (1.00)
	Conjugate + Hcp1	66 (1.17)
	Conjugate + Hcp1 + AhpC	200,316 (1.49)
	668 *ΔilvI*	60 (1.15)
OPS	PBS	55 (1.12)
	Adjuvant	50 (1.00)
	Conjugate + Hcp1	174 (1.43)
	Conjugate + Hcp1 + AhpC	276 (1.44)
	668 *ΔilvI*	2,660 (1.54)
Bm FMH	PBS	57 (1.14)
	Adjuvant	50 (1.00)
	Conjugate + Hcp1	8,063 (1.89)
	Conjugate + Hcp1 + AhpC	53,283 (2.78)
	668 *ΔilvI*	764 (1.83)

a Wells were coated with the indicated antigen.

b n = 5 animal sera per group.

c Values represent the GM and GSE IgG titers.

The total IgG responses of the mice 4 weeks after the last vaccine dose (day 65) are presented in Table 3. For most of the antigens, the titers were similar to those determined 6 days after vaccination. The mean day 65 anti-BpK titer of the 668 Δ*ilvI*- vaccinated mice were 2-fold higher than that of day 44, though not significantly different. Similar to the day 44 findings, the 668 Δ*ilvI* day 65 mean titer to inactivated Bm FMH was 8-fold lower than that to BpK (4,222 vs. 33,513 reciprocal titer, respectively). Also, the 668 Δ*ilvI* anti-Bm FMH level was similar to, and not significantly different from, the anti-Bm FMH responses of the two subunit vaccines. As expected, the two subunit vaccines exhibited increases in titers to Hcp1 at day 65. Notably, the day 65 anti-Hcp1 titer of the Conjugate + Hcp1 group was nearly 6-fold greater than that at day 44 (*p* = 0.0079), although relative to day 44, the increase in anti-CPS titers for the two subunit vaccines on day 65 was negligible. Sera from the LAV group again exhibited a deficiency in humoral immunity to Hcp1 and CPS antigens. The elevated anti-OPS IgG levels of the 668 Δ*ilvI* group observed with the day 44 sera were accentuated for the day 65 sera. Whereas, the subunit vaccine groups had anti-OPS titers that were only slightly higher than the controls (*p* = 0.024 and 0.119, for subunit vaccines without/with AhpC, respectively) (Table 3). Lastly, the Conjugate + Hcp1 + AhpC group was again uniquely reactive to AhpC antigen and produced very high anti-AhpC titers.

**Table 3:** Total IgG and subclass responses 28 days post-vaccination (day 65) in C57BL/6 mice.

**Antigen^a^**	**Groups^b^**	**IgG^c^**	**IgG1**	**IgG2c**	**IgG2c/IgG1 ratio**
BpK	PBS	52 (1.08)	50 (1.00)	50 (1.00)	1.00
	Adjuvant	50 (1.00)	50 (1.00)	50 (1.00)	1.00
	Conjugate + Hcp1	3,055 (2.49)	9,670 (3.34)	553 (2.36)	0.06
	Conjugate + Hcp1 + AhpC	3,510 (2.57)	17,521 (3.30)	1,106 (1.77)	0.06
	668 *ΔilvI*	33,513 (1.76)	16,783 (2.06)	7,699 (2.41)	0.46
Hcp1	PBS	100 (1.26)	ND^d^ –	ND –	–
	Adjuvant	79 (1.21)	ND –	ND –	–
	Conjugate + Hcp1	844,485 (1.36)	53,031 (1.96)	559,801 (1.79)	10.56
	Conjugate + Hcp1 + AhpC	92,625 (1.64)	22,146 (3.37)	88,442 (1.22)	4.00
	668 *ΔilvI*	83 (1.20)	ND –	ND –	–
CPS	PBS	50 (1.00)	ND –	ND –	–
	Adjuvant	52 (1.08)	ND –	ND –	–
	Conjugate + Hcp1	84,049 (2.55)	193,399 (2.80)	23,193 (2.16)	0.12
	Conjugate + Hcp1 + AhpC	66,499 (1.81)	153,501 (2.38)	13,300 (1.68)	0.09
	668 *ΔilvI*	50 (1.00)	ND –	ND –	–
AhpC	PBS	50 (1.00)	ND –	ND –	–
	Adjuvant	50 (1.00)	ND –	ND –	–
	Conjugate + Hcp1	63 (1.21)	ND –	ND –	–
	Conjugate + Hcp1 + AhpC	306,033 (1.49)	769,936 (0.43)	5,554 (1.73)	0.01
	668 *ΔilvI*	50 (1.00)	ND –	ND –	–
OPS	PBS	66 (1.22)	ND –	ND –	–
	Adjuvant	50 (1.00)	ND –	ND –	–
	Conjugate + Hcp1	166 (1.35)	ND –	ND –	–
	Conjugate + Hcp1 + AhpC	159 (1.47)	ND –	ND –	–
	668 *ΔilvI*	26,642 (1.61)	9,204 (2.68)	4,422 (2.36)	0.48
Bm FMH	PBS	76 (1.29)	ND –	ND –	–
	Adjuvant	50 (1.00)	ND	ND –	–
	Conjugate + Hcp1	5,303 (1.70)	5,807 (3.86)	1,106 (2.29)	0.19
	Conjugate + Hcp1 + AhpC	8,418 (2.09)	9,233 (3.66)	665 (1.32)	0.07
	668 *ΔilvI*	4,222 (1.57)	419 (1.79)	579 (2.40)	1.38

a Wells were coated with the indicated antigen.

b n = 5 animal sera per group.

c Values represent GM and GSE IgG titers.

d nd = not determined since total IgG titer was ≤ limit of detection (~50).

In addition to total IgG, subclass IgG1 and IgG2c antibody responses were assessed with the sera obtained before challenge (day 65). Both of the conjugate subunit vaccines induced IgG1 titers to inactivated BpK and Bm FMH antigens that were much higher than the corresponding IgG2c antibody levels (Table 3). In contrast, the 668 Δ*ilvI*-induced IgG1 and IgG2c subclass antibody titers to these whole cells were not significantly different (Table 3); and not surprisingly, the LAV-vaccinated mice exhibited a higher IgG2c/IgG1 ratio to BpK than did subunit-vaccinated mice. The 668 Δ*ilvI* IgG titers to Hcp1 and CPS were too low for evaluation, but the anti-CPS antibody subclass ratios of the subunit vaccines were highly Th2 skewed (as were the anti-AhpC IgG2c responses from the Conjugate + Hcp1 + AhpC vaccine). Conversely, the anti-Hcp1 IgG2c/IgG1 ratios of the subunit vaccines exhibited Th1 polarity, with IgG2c levels that were four-fold (Conjugate + Hcp1 + AhpC) and ten-fold (Conjugate + Hcp1) higher than the IgG1 titers. This response had been observed previously in vaccinated C57BL/6 mice, i.e., Conjugate + Hcp1 vaccine induced a moderately Th1 polar subclass response, with an IgG2c/IgG1 ratio of 2.2. Finally, the titers of the subunit vaccines to the OPS antigen were too low to be evaluated in the subunit vaccines as the mice were not vaccinated with the OPS antigen and the low titers could be a consequence of cross-reactivity between O-acetyl groups on CPS and OPS. However, the antibody subclass ratio of 668 Δ*ilvI* suggested a balanced Th1/Th2 response to OPS (IgG1 and IgG2c titers not significantly different). Thus, significant differences were observed in the relative IgG1 and IgG2c antibody responses, depending on the vaccine group and the antigen examined.

#### Cell-mediated immune responses: Cytokines produced by splenocytes

Samples from vaccinated animals were collected at various time-points pre- and post-challenge for ELISpot and Luminex restimulation assays (spleen cells) and for cytokine profiling (spleen and lung homogenates). Six days after the last vaccine dose (day 44), spleens were removed from mice in each group and the splenocytes stimulated with Hcp1 or AhpC. ELISpot was used to assess the number of IFN-γ secreting cells. Table 4A shows the highest number of IFN-γ secreting splenocytes after Hcp1 stimulation were produced by the Conjugate + Hcp1 vaccine group (49 SFC), followed by the Conjugate + Hcp1 + AhpC vaccine group (39 SFC); both were significantly greater than the PBS control SFCs; the number of SFCs after Hcp1 stimulation of the 668 Δ*ilvI* group did not differ from those of the controls (Table 4A). The addition of AhpC to the Conjugate + Hcp1 vaccine formulation appeared to diminish the response to Hcp1; however, the difference was not statistically significant (Table 4A). The low levels of IFN-γ secreting splenocytes after Hcp1 stimulation in the 668 Δ*ilvI* group suggests that this vaccine produced relatively low levels of Hcp1. Table 4B shows the highest number of IFN-γ secreting splenocytes after AhpC stimulation were exhibited by the Conjugate + Hcp1 + AhpC vaccine group (1,010 SFC). There was some residual activation in the Conjugate + Hcp1 vaccinated group (93 SFC) after stimulation with AhpC, and it was greater than that of the Adjuvant controls (*p* = 0.0084). The small number of IFN-γ secreting splenocytes after AhpC stimulation in the 668 Δ*ilvI* group was not significantly different from that of the PBS control, and suggests that these vaccines produced relatively low levels of AhpC. The relative differences in numbers of IFN-γ -producing splenocytes after Hcp1 and AhpC stimulation were congruent to the differences in IFN-γ concentrations in splenocyte culture supernatants as determined in luminex assays, as described below.

**Table 4:** IFNγ-secreting splenocytes detected after stimulation 6 days post-vaccination (day 44) with Hcp1 or AhpC.

**Parameter**	**PBS**	**Adjuvant**	**Conjugate + Hcp1**	**Conjugate + Hcp1 + AhpC**	**668 *ΔilvI***
**A—Stimulation with 5 μg Hcp1**					
Spot forming cells (SFC)^a^	4 (1.25)	7 (1.29)	49 (2.00)	39 (1.50)	2 (1.38)
vs. Adjuvant^b^	0.2732				
vs. Conjugate + Hcp1^b^	0.0231	0.0426			
vs. Conjugate + Hcp1 + AhpC^b^	0.003	0.008	0.7989		
vs. 668 Δ*ilvI*^b^	0.1082	0.027	0.0075	0.0006	
**B—Stimulation with 5 μg AhpC**					
Spot forming cells (SFC)^a^	11 (1.18)	12 (1.46)	93 (1.57)	1,010 (1.33)	8 (1.46)
vs. Adjuvant^b^	0.8609				
vs. Conjugate + Hcp1^b^	0.0064	0.0084			
vs. Conjugate + Hcp1 + AhpC^b^	<0.0001	<0.0001	0.0032		
vs. 668 Δ*ilvI*^b^	0.4079	0.4266	0.003	<0.0001	

a The splenocyte response was assessed as spot forming cells (SFC), adjusted to 10^6^ cells per well. Values represent GM and GSE.

b The groups (n = 5) were compared using the Welch's t-test on log-transformed data; p values are shown for the five sets of comparisons.

Multiplex cytokine analysis was performed with splenocytes obtained 6 days post-vaccination (day 44) and restimulated. After Hcp1 stimulation, cytokine production was enhanced the greatest overall for the Conjugate + Hcp1 group, followed in descending order by responses of the Conjugate + Hcp1 + AhpC, and 668 Δ*ilvI* groups, as shown by the profiles of cytokine concentration and fold-increase relative to PBS; the color scale indicates the extent of increase (green) or decrease (red) relative to the control value (Table 5A). In the subunit vaccinated mice, four cytokines were stimulated to the greatest extent compared to the controls: MCP-3/CCL7, MCP-1/CCL2, IP-10/CCX-10, and IFN-γ (*p* < 0.0001–0.0497). These four cytokines were not significantly increased compared to the PBS group in 668 Δ*ilvI*, and MCP-3 was produced at significantly reduced levels, *p* < 0.0001. IL-13 levels were highly elevated in all three vaccine groups compared to the PBS control, however it was also stimulated in the Adjuvant control. IL-23 was upregulated uniquely in the 668 Δ*ilvI* splenocytes (*p* < 0.0001), while Eotaxin/CCL11 was down regulated in all three vaccine groups.

**Table 5:**
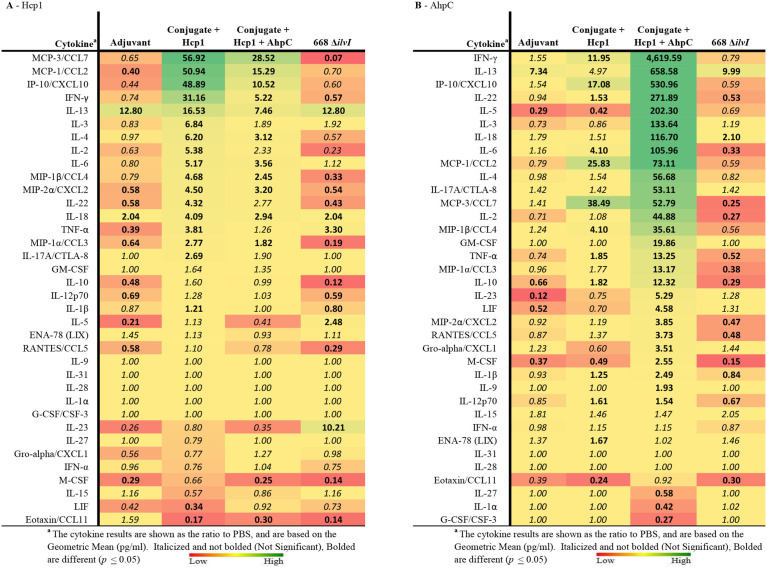
The fold change relative to PBS group in cytokine responses in splenocytes obtained 6 days after vaccination (Day 44) and stimulated with Hcp1 or AhpC antigen.

AhpC stimulated a higher overall cytokine response relative to Hcp1 stimulation, and the levels were greatest for the Conjugate + Hcp1 + AhpC group, followed in descending order by responses of the Conjugate + Hcp1, and 668 Δ*ilvI* groups, as shown by comparison of cytokine levels (pg/ml) and the fold-increase relative to PBS (Table 5B). Cytokine levels were downregulated most often and to the greatest extent in the 668 Δ*ilvI* group; however, IL-13 was again upregulated, much like after Hcp1 stimulation, in the 668 Δ*ilvI* group. The level of IFN-γ expressed by the AhpC-stimulated Conjugate + Hcp1 +AhpC group was notably much greater than that of the other 36 cytokines assayed (*p* < 0.0001). Thus, results from the multiplex analyses support the inferences from the ELISpot results, described above. Unlike the subunit vaccine responses, the levels of many cytokines produced by the 668 Δ*ilvI* group were reduced relative to the PBS control.

Splenocytes were also collected from the mice on day 65 and cytokine profiles determined. After Hcp1 stimulation, the relative findings for the vaccine groups were similar to those assayed post-vaccination (day 44), however there was a decrease in the overall cytokine response from day 44 (6 days post boost) to day 65 (Supplementary Table 1A). For instance, analogous to the day 44 findings, IFN-γ levels were significantly enhanced over the controls in the two subunit vaccines (*p* ≤ 0.0022), although differences were not as great as compared to day 44. This was again not elevated in the 668 Δ*ilvI* group. Also, the MCP-3/CCL7 levels were again notably elevated in only the subunit groups compared to both control groups (Supplementary Tables 1A, 2). As before, the cytokine stimulation induced by Hcp1 of the 668 Δ*ilvI* group splenocytes was reduced for other cytokines such as IFN-γ, IL-3, IL-2, TNF-α, and IL-10. Of interest, IL-31, a Th2-associated cytokine, was upregulated to a significant extent (4.4–11.2-fold) compared to the controls in all vaccine groups (*p* = 0.02–0.0003). The response after AhpC stimulation was once more greater in magnitude overall than that after Hcp1 stimulation (Supplementary Table 1B); however only the AhpC-stimulated cytokines expressed by the Conjugate + Hcp1+AhpC group were elevated significantly compared to the PBS control, and IFN-γ was the most notably upregulated (>1,200-fold greater than PBS, *p* = 0.0001). Also, IL-13, IL-17A, IL-5, IP-10, IL-4, and numerous other Th1- and Th2-associated cytokines were markedly increased relative to PBS in Conjugate + Hcp1 + AhpC vaccinated mice, *p* < 0.0001 (Supplementary Tables 1, 2).

#### Cell-mediated immune responses: Cytokines produced in tissue homogenate supernatants

Homogenates of lungs and spleens collected on day 44 and 65 were prepared and supernatants assessed directly for cytokine responses. For lung and spleen homogenates at both time-points, the extent and relative amounts of cytokines expressed were generally similar between the vaccine and control groups (data not shown). For the samples collected 6 days post-vaccination (day 44), there were small but significant differences in levels relative to PBS (*p* < 0.05) for some of the cytokines. However, none of the differences were greater or lesser by at least 2-fold (i.e., >2.0x or <0.5x) relative to the PBS and Adjuvant controls. The day 65 lung and spleen samples from the 668 Δ*ilvI* vaccinated group had the largest number of cytokines that were moderately upregulated relative to PBS (Supplementary Tables 2A,B). This situation was possibly related to prolonged antigen retention associated with live vaccine growth and persistence relative to its subunit vaccine counterparts or differences in antigen trafficking based on the type of antigen and site of administration (O'Callaghan et al., 1988; Fuller et al., 2000; Johansen et al., 2008).

**Figure 4:**
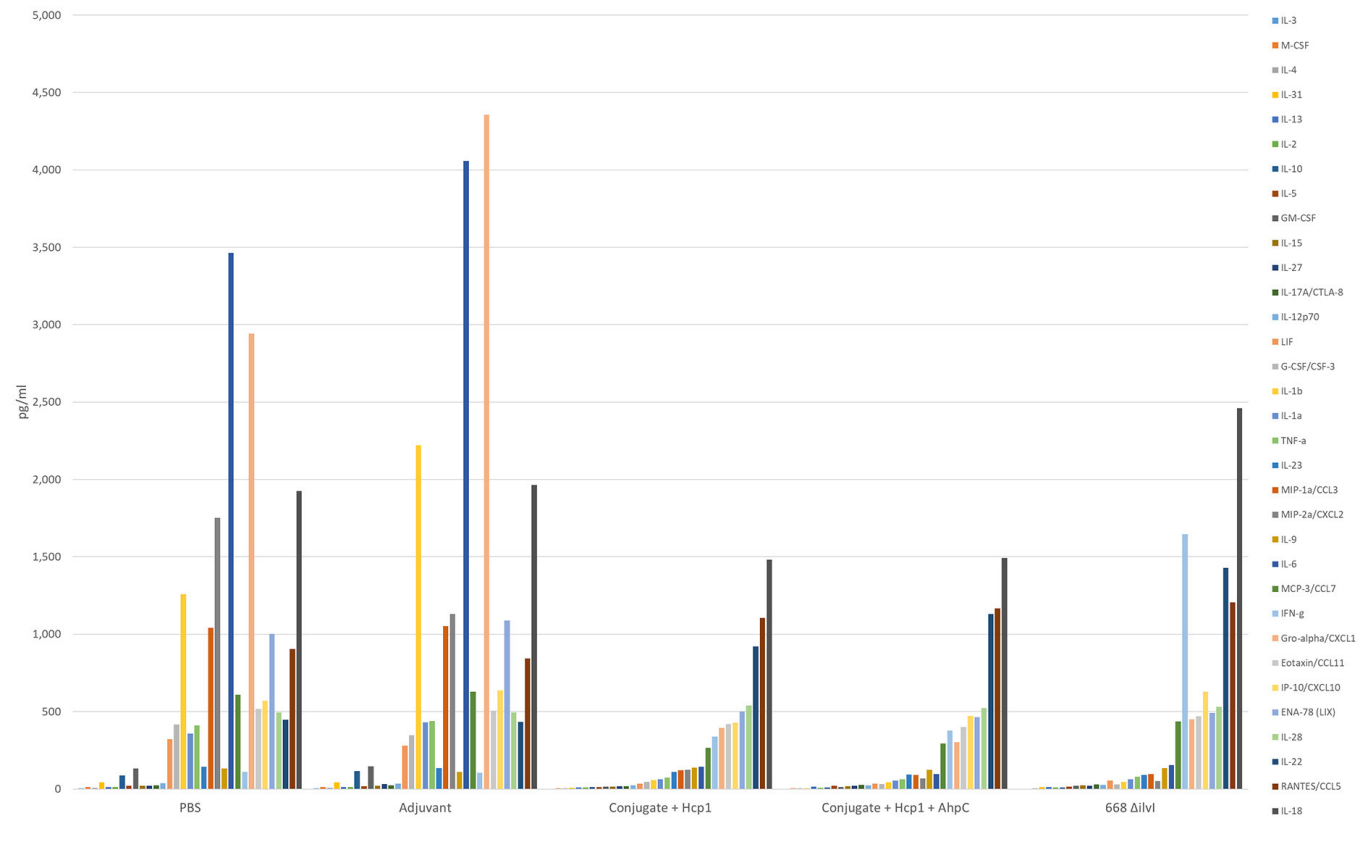
Cytokine analysis of lung homogenates from vaccinated mice collected 3 days after challenge with a 3.4 LD_50_ dose of *Bp* K96243. Cytokine levels are depicted as pg/ml homogenate supernatant.

Cytokine profiles of lung and spleen homogenates from all groups of mice were also assessed at 3 days post-challenge with *Bp* K96243. All the differences in cytokines levels relative to the control groups cited below were significant, p <0.05, as detailed in Table 6, and illustrated in Figure 4. The Luminex results indicate that, relative to the PBS and Adjuvant controls, 13 cytokines (IL-6, IL-1α and 1β, G-CSF, GM-CSF, M-CSF, IL-10, Gro-alpha, MIP-1α and 2α, TNF-α, LIF, and IL-31) were significantly suppressed in all vaccinated groups (Figure 4, Table 6). However, IFN-γ and IL-22 were substantially upregulated in all three vaccine groups, especially 668 Δ*ilvI*. IFN-γ was increased 3.0- to 3.4-fold in the Conjugate + Hcp1 subunit groups and 14.8-fold in 668 Δ*ilvI* homogenates; and IL-22 was significantly elevated compared to PBS in 668 Δ*ilvI* (3.2-fold), as shown in Figure 4 and Table 6. Overall, a more controlled cytokine response stimulated by infection appeared to mirror the better protection (compared to the controls) afforded by the subunit and 668 Δ*ilvI* vaccine groups against inhalational melioidosis.

**Table 6:**
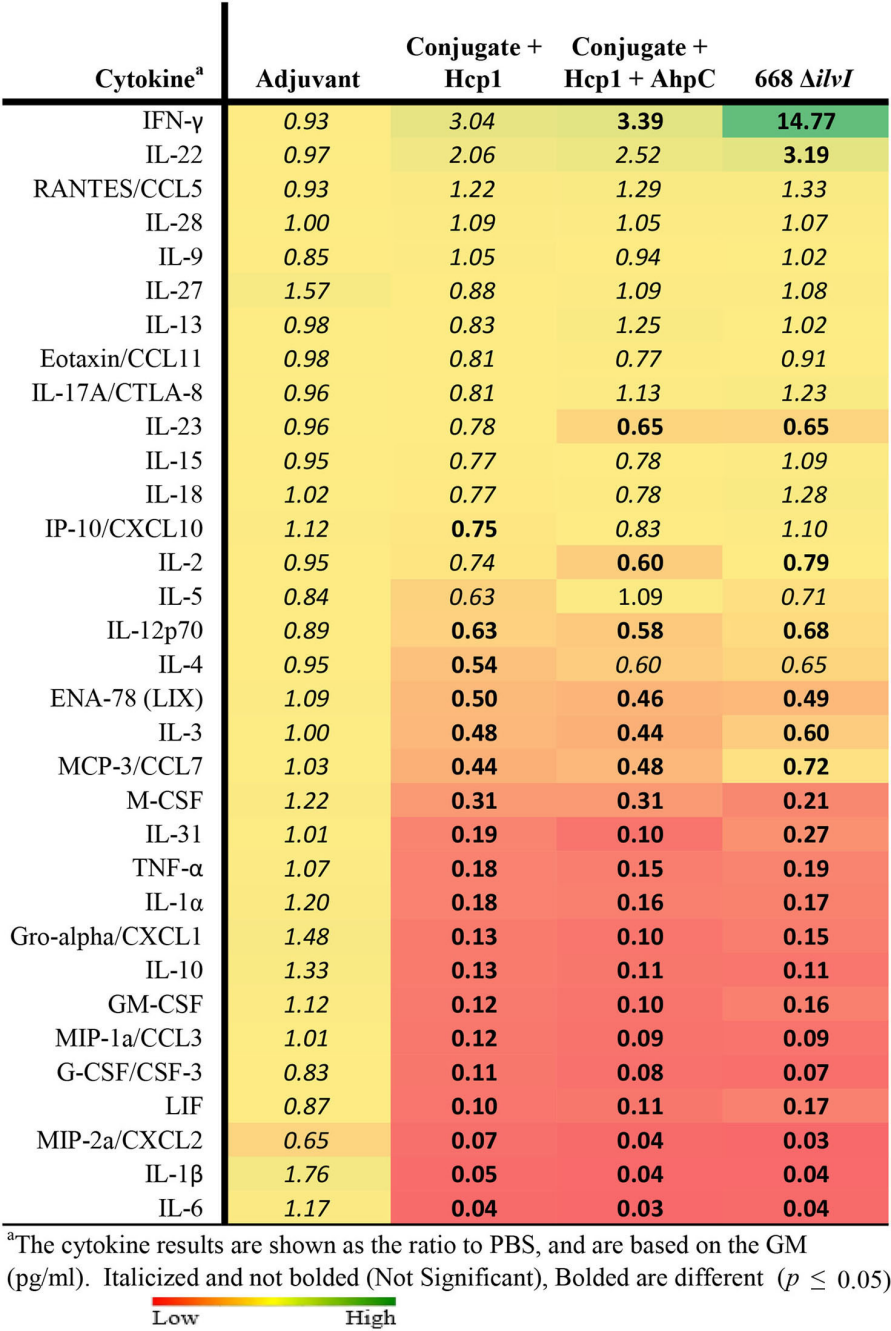
Fold change relative to PBS in cytokine responses in lung homogenates from mice three days post-challenge with *Bp* K96243.

The cytokine profiles of spleen homogenates from mice 3 days after challenge were also examined. In all three vaccine groups, cytokine G-CSF/CSF-3 was highly suppressed (*p* ≤ 0.011); and IL-10 was significantly down-regulated in the Conjugate + Hcp1 + AhpC and 668 Δ*ilvI* groups (*p* = 0.02–0.04) and reduced but not significantly in Conjugate + Hcp1 mice. In contrast to the lung homogenate profile, IFN-γ was significantly suppressed in both subunit groups, Conjugate + Hcp1 (*p* = 0.03) and Conjugate + Hcp1 + AhpC, *p* = 0.007, but not significantly reduced in the 668 Δ*ilvI* group (Supplementary Figure 3, Supplementary Table 3). Thus, the spleen post-challenge cytokine data again appeared to be associated with the substantial protection afforded by the vaccines as evidenced by better control of the infection-induced cytokine release than was observed in the control mice.

The relative changes in cytokine levels for pre-challenge (day 65) compared to day 3 post-challenge lung and spleen samples were determined, as illustrated in Table 7 (lung) and spleen (Supplementary Table 4). The changes in lung cytokines revealed increased levels of IFN-γ for post-challenge relative to pre-challenge in all five mice groups but was most pronounced in the three vaccine groups. The increases ranged from 20- to 26-fold for the Adjuvant and PBS groups (not significant), 86- to 87-fold for the two subunit vaccine groups (*p* = 0.0496 for Conjugate + Hcp1 + AhpC), and nearly 200-fold for 668 Δ*ilvI* (*p* = 0.004). Although cytokines IL-18 and IL-22 were uniformly more elevated in all five groups on day 3 post-challenge compared to pre-challenge, the increases were not statistically different (Table 7). However, for many of the cytokines examined, the cytokine levels in the control group were highly increased in the post-compared to pre-challenge homogenates, in contrast to the lesser, more controlled increases (or decreases) in the subunit- and LAV-vaccinated group post-challenge samples. These findings were observed especially for Gro-α/CXCL1, MIP-1α, IL-1β, IL-1α, TNFα, IL-6, M-CSF, IL-10, GM-CSF, and IL-31. The pattern of changes in cytokines detected in the spleen homogenates of the vaccinated and control mouse groups was similar; and the fold-changes in the before-exposure relative to after-exposure levels were generally much greater for the PBS and Adjuvant control groups than for the subunit and 668 Δ*ilvI* vaccine groups. The results suggest that the unvaccinated mice responded to virulent challenge with an uncontrolled cytokine response, which was better modulated in all three vaccine groups; exceptions included enhanced production by the latter groups of a few potentially protective cytokines, especially IFN-γ and IL-22.

**Table 7:**
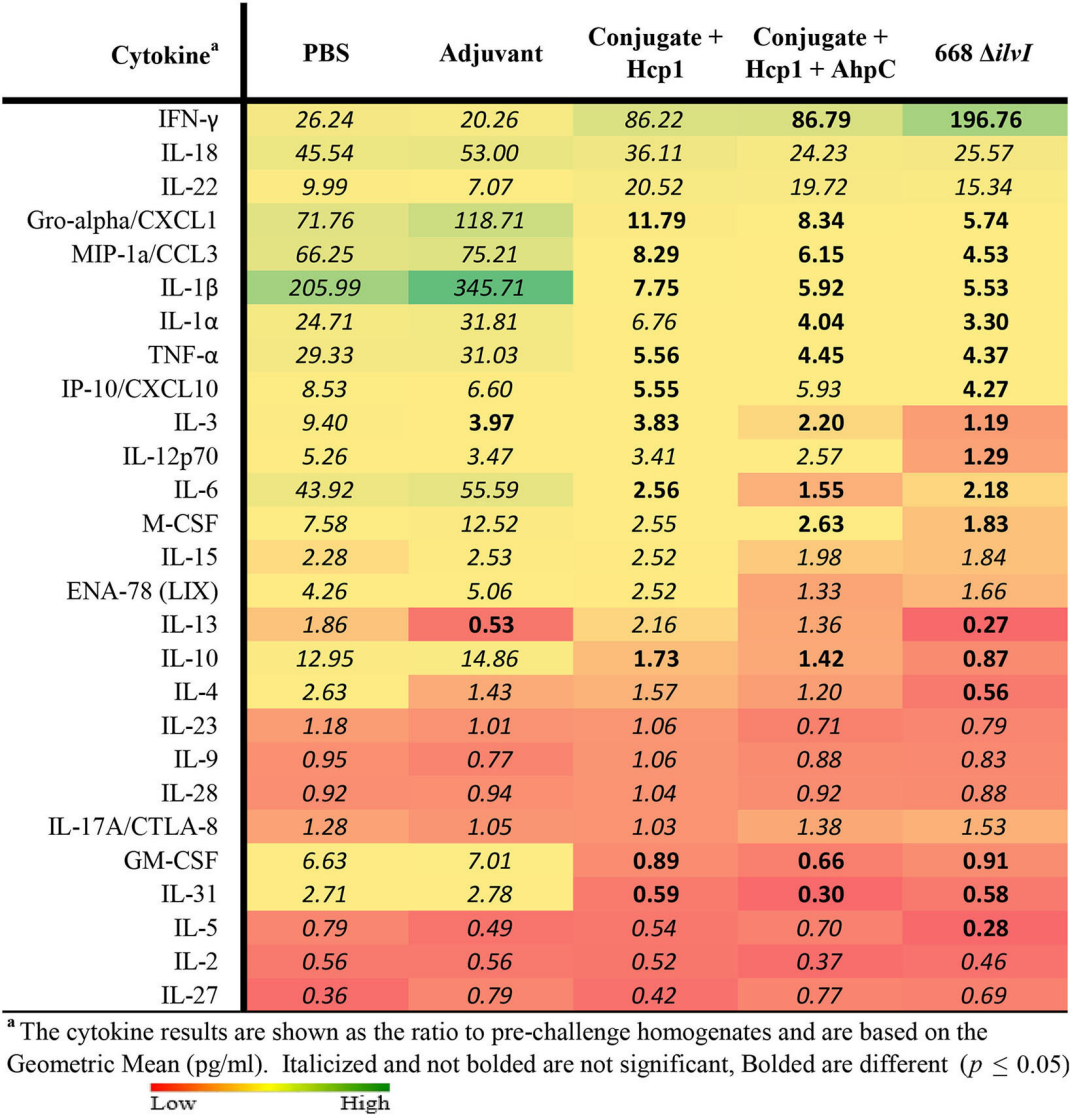
Fold change in cytokine levels post-challenge with *Bp* K96243 in lung homogenates relative to day 65 (pre-challenge) samples.

### Comparison of melioidosis vaccines against *B. mallei* FMH and *B. pseudomallei* strain MSHR5855 in a C57BL/6 mouse aerosol model of infection

In further studies, we compared the protection afforded by two of the candidates, 668 Δ*ilvI* and Conjugate + Hcp1, against infection by *Bm* strain FMH and the highly virulent *Bp* stain MSHR5855. The vaccines were prepared and administered to C57BL/6 mice in accordance with the scheme described for the preceding study. We chose to use the MSHR5855 strain in these studies so that we could evaluate the vaccine efficacy in a clinical isolate from Northern Australia with documented neurological involvement in non-human primates (Trevino et al., 2021). This is in contrast with the K96243 isolate, a common laboratory stain that was originally a clinical isolate from Thailand.

#### Efficacy

**Figure 5:**
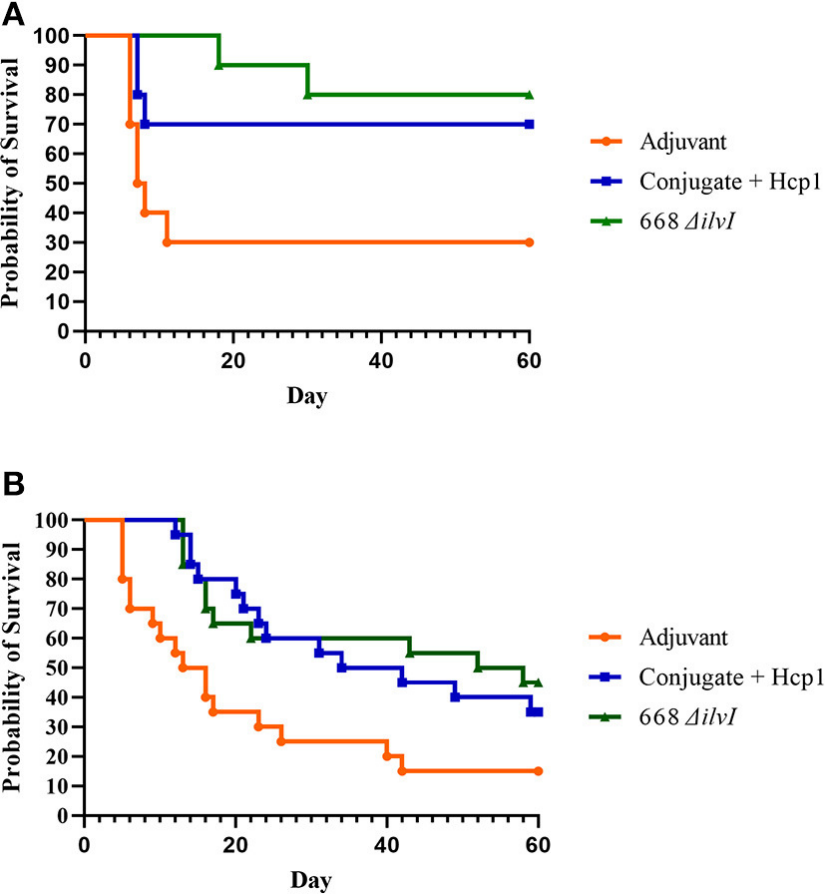
**(A)** Survival curves of vaccinated and control C57BL/6 mice (*n* = 20/group) challenged with *Bm* FMH by the aerosol route. Mice were exposed to 4 LD_50_ or 11 LD_50_ in two separate aerosol exposures of ten mice per group. **(B)** Survival curves of vaccinated and control C57BL/6 mice (*n* = 20/group) challenged with *Bp* MSHR5855 by the aerosol route. Mice were exposed to inhaled doses of 11 LD_50_ or 12 LD_50_ in two separate aerosol exposures of ten mice per group.

Two aerosol exposures were done for both challenge strains, and the survival data of the two exposures combined (*n* = 20). The inhaled doses of *Bm* FMH were 4 LD_50_ and 11 LD_50_ (mean 7.5 LD_50_); and for *Bp* MSHR5855 challenges, the inhaled doses were 11 LD_50_ and 12 LD_50_. The subunit and LAV both afforded significant protection compared to the Adjuvant controls against lethal aerosol exposure to *Bm* FMH as determined by the survival rates and mean TTMs (Figure 5A, Supplementary Figure 4A). By the study endpoint (day 60 post-challenge), 85 and 80% of the 668 Δ*ilvI*- and Conjugate + Hcp1-vaccinated mice, respectively, survived the mean exposure dose whereas 25% of the controls survived [Figure 5A (*p* = 0.0012 and 0.0038, respectively)]. The survival rates diverged early post-exposure. The survival rate of the 668 Δ*ilvI* mice was greater than that of the Adjuvant controls by day 7 (*p* = 0.0108), and at day 21 post-challenge, the 668 Δ*ilvI* and Conjugate + Hcp1 group rates were both increased compared to that of the controls (*p* < 0.0001 and 0.0012). The TTMs were also increased for the vaccinated mice, i.e., 26.5 days for 668 Δ*ilvI*, 17.2 days for Conjugate + Hcp1, and 6.1 days for the control group (*p* < 0.0001, LAV, and *p* = 0.0003, subunit vaccine) as shown in Supplementary Figure 4A. The mean TTM of the 668 Δ*ilvI*-vaccinated mice was greater than that of the subunit-vaccinated mice, but the difference was not significant. The vaccines appeared to be partially protective against the virulent *Bp* MSHR5855 strain at the 60 day endpoint. The survival rates were 40% (668 Δ*ilvI*) and 35% (Conjugate + Hcp1) compared to the control survival rate of 15%, but were not significantly different (Figure 5B). Nonetheless, the survival rate at an earlier time post-exposure (day 21) for the Conjugate + Hcp1-vaccinated mice was greater than that of the Adjuvant controls (*p* = 0.056). Also, the TTMs at the study endpoint of the vaccinated mice were longer than that of the controls (Supplementary Figure 4B), i.e., 24.1 days for 668 Δ*ilvI*, 27.5 days for Conjugate + Hcp1, and 15.1 days for the control group, with p=0.018 for both vaccine groups.

#### Bacteriology

##### *B. mallei* FMH-challenged mice

**Figure 6:**
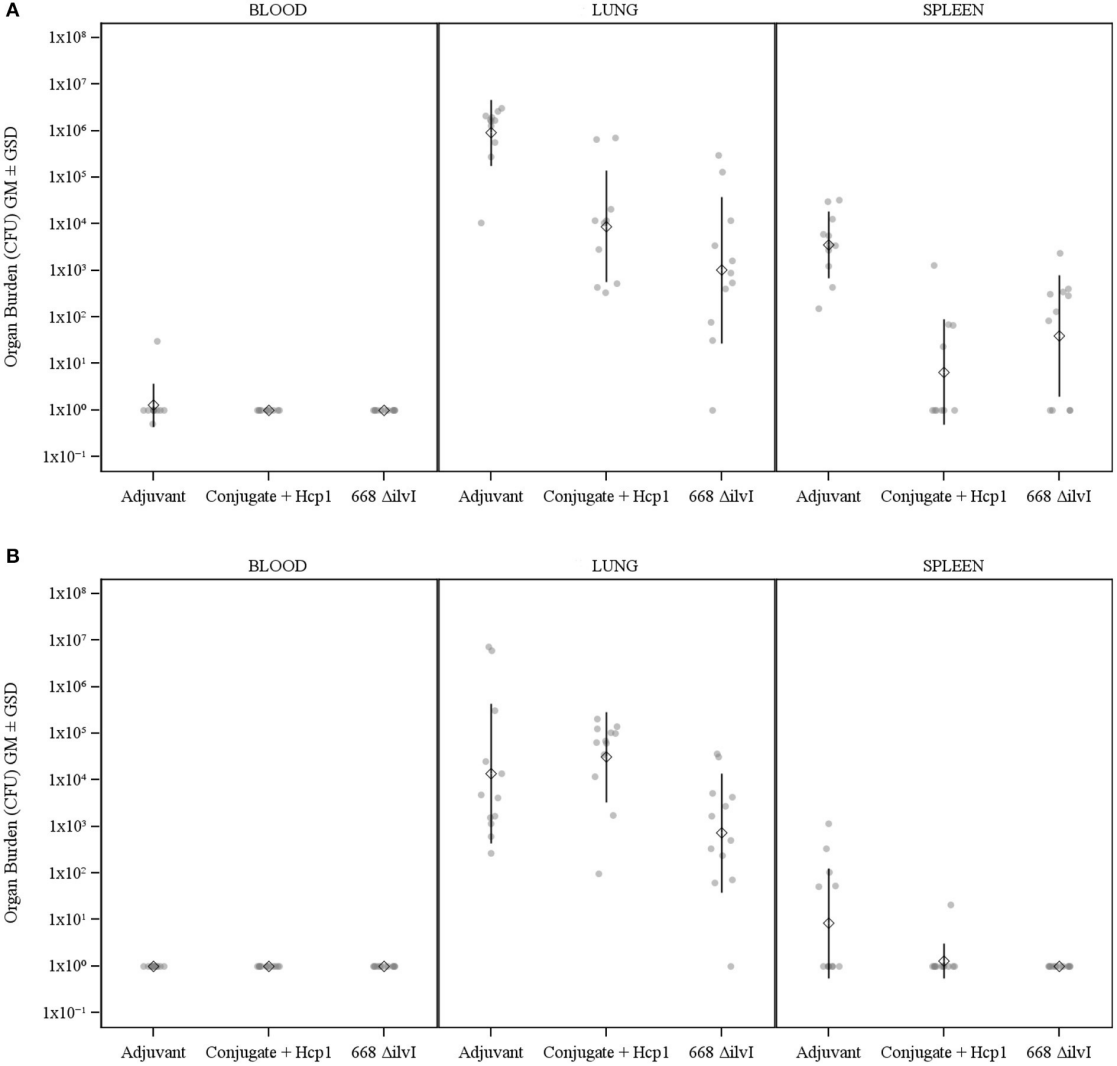
**(A)** The recovery of bacteria as determined by CFU counts from blood, spleens and lungs of C57BL/6 mice 3 days post-challenge with *Bm* FMH. The combined results for both challenges are shown (*n* = 10–11 mice/vaccine group). **(B)** The recovery of bacteria as determined by CFU counts from blood, spleens and lungs of C57BL/6 mice 3 days post-challenge with *Bp* MSHR5855. The combined results for both challenges are shown (*n* = 12 mice/group). The left axis represents CFU/ml (Blood) or CFU/g (Lung and Spleen). The individual points represent one animal, and the baseline points indicate the remaining survivors with no detectable CFU. The diamond-shaped symbol and vertical line are the GM and GSD, respectively. The limits of detection were ~10 CFU/ml (blood) and 5 CFU/g (organs).

The number of viable bacteria present in the blood, spleens, and lungs of animals per vaccine group were determined on day 3 post-challenge. For the *Bm* FMH-challenged groups, the only mice with detectable bacteria in the bloodstream were two Adjuvant control mice (10 and 30 CFU/ml) as shown in Figure 6A. The lung samples were the most heavily colonized in all groups, i.e., the controls had a mean of 8.8x10^5^ CFU/g and the vaccinated mice had a >2 log lower burden of bacteria, 8.8 × 10^3^ (Conjugate + Hcp1) and 1.1 × 10^3^ CFU/g (668 Δ*ilvI*), *p* = 0.0008 and *p* < 0.0001, respectively. The lung counts of the two vaccine groups were not significantly different. Similarly, whereas the controls harbored a mean of 3.5 × 10^3^ CFU/g spleen, only 6.5 and 39 CFU/g spleen were recovered from the spleens of the Conjugate + Hcp1- and 668 Δ*ilvI*- vaccinated mice (*p* < 0.0001 and *p* = 0.0002, respectively); mean spleen counts for the two vaccine groups were again not significantly different, as shown in Figure 6A. The number of viable *Bm* FMH present in these tissues of mice that survived 60 days after aerosol exposure was also determined and most survivors had no recoverable CFU in lungs; however, spleens were still positive in some mice of all groups (Supplementary Table 5).

##### *B. pseudomallei* MSHR5855-challenged mice

The bacterial burden in the blood, lungs, and spleens of mice sampled at day 3 post-challenge with *Bp* MSHR5855 was also examined (Figure 6B). None of the mice had detectable bacteria in the blood. As observed for *Bm*, the lung samples were again the most heavily colonized; however, whereas the Adjuvant controls and Conjugate + Hcp1 vaccinated mice had means of 1.4 × 10^4^ and 3.2 × 10^4^ CFU/g lung respectively, the 668 Δ*ilvI* vaccinated mice had a nearly two log lower mean count, 7.2 × 10^2^ CFU/g lung, *p* = 0.043 and *p* = 0.0011, respectively. The spleen burden was less than that of the lungs, probably reflecting the time lag for systemic dissemination of the bacteria from the lungs. There were no detectable organisms in all twelve 668 Δ*ilvI*-vaccinated mice and all except one Conjugate + Hcp1-vaccinated mouse (21 CFU/g); five of the controls had low CFU levels with a mean of 8.3 CFU/g, significantly greater than that for both groups of vaccinated animals (*p* = 0.0373). As observed for the *Bm* challenge, these early post-challenge CFU data appeared to parallel the greater early phase survival after *Bp* MSHR5855 challenge of the vaccinated mice (especially those receiving 668 Δ*ilvI*) compared to controls.

The number of viable *Bp* MSHR5855 present in the tissues of survivors at the end of the 60-day post-challenge period was assessed. There were no viable bacteria detected in the blood and spleen of any survivor of the three groups. The lungs revealed low levels in a few mice, but CFU were not recovered from most of the mice. Due to the low overall recovery of *Bp* MSHR5855 from tissues, there were no statistical differences. However, we approached achieving sterile immunity at 60 days in MSHR5855 challenged mice, with no CFU recovered from blood and spleen of any vaccinated mice. CFU were only detected in lungs of 2 of 7 Conjugate + Hcp1 survivors and in 1 of 8 remaining 668 Δ*ilvI* mice.

#### Cell-mediated immune responses

Cytokine profiles of lung and spleen homogenates from the mice were determined by Luminex analysis on day 3 post-challenge with *Bm* FMH and *Bp* MSHR5855, as shown in Tables 8, 9. The Luminex-based cytokine profiles of lung and spleen homogenates from all three groups of mice were assessed at 3 days post-challenge with 11 LD_50_ (431 CFU) of *Bp* MSHR5855 and 11 LD_50_ (1.12 × 10^4^ CFU) of *Bm* FMH. For the animals challenged with *Bm* FMH, there was a noticeable increase in IL-17A, IFN-γ, IL-22, IL-4, IL-3, and IL-5 in the lungs in both vaccine groups. The response was greater in the Conjugate + Hcp1-vaccinated mice than the 668 Δ*ilvI*-vaccinated mice (Table 8 and data not shown); the color scale indicates the extent of increase (green) or decrease (red) relative to the control value. IL-1β, G-CSF, MIP-2, MIP-1α, and MIP-1β were down-regulated in both vaccine groups, but these cytokines were even more down-regulated in the 668 Δ*ilvI* relative to Conjugate + Hcp1 vaccinated group. There was very limited upregulation of the cytokines detected in the spleen homogenates at 3 days post-challenge with *Bm* FMH, with no cytokines transcending a two-fold increase, but G-CSF, IFN-γ, IL-5, and GM-CSF were downregulated in both vaccine groups based on at minimum a two-fold reduction (*p* ≤ 0.5; Supplementary Table 6).

**Table 8:**
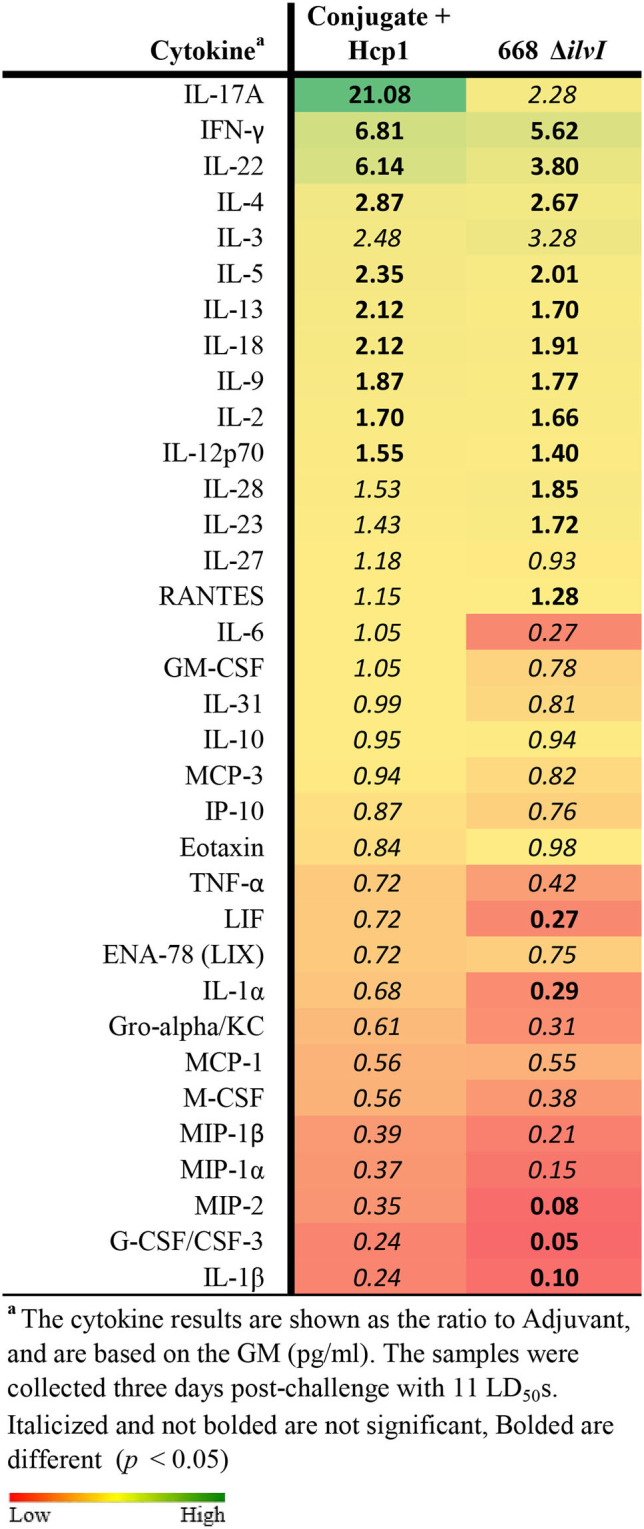
Fold change on day 3 post-challenge relative to Adjuvant group of lung homogenates from vaccinated mice challenged with *Bm* FMH.

**Table 9:**
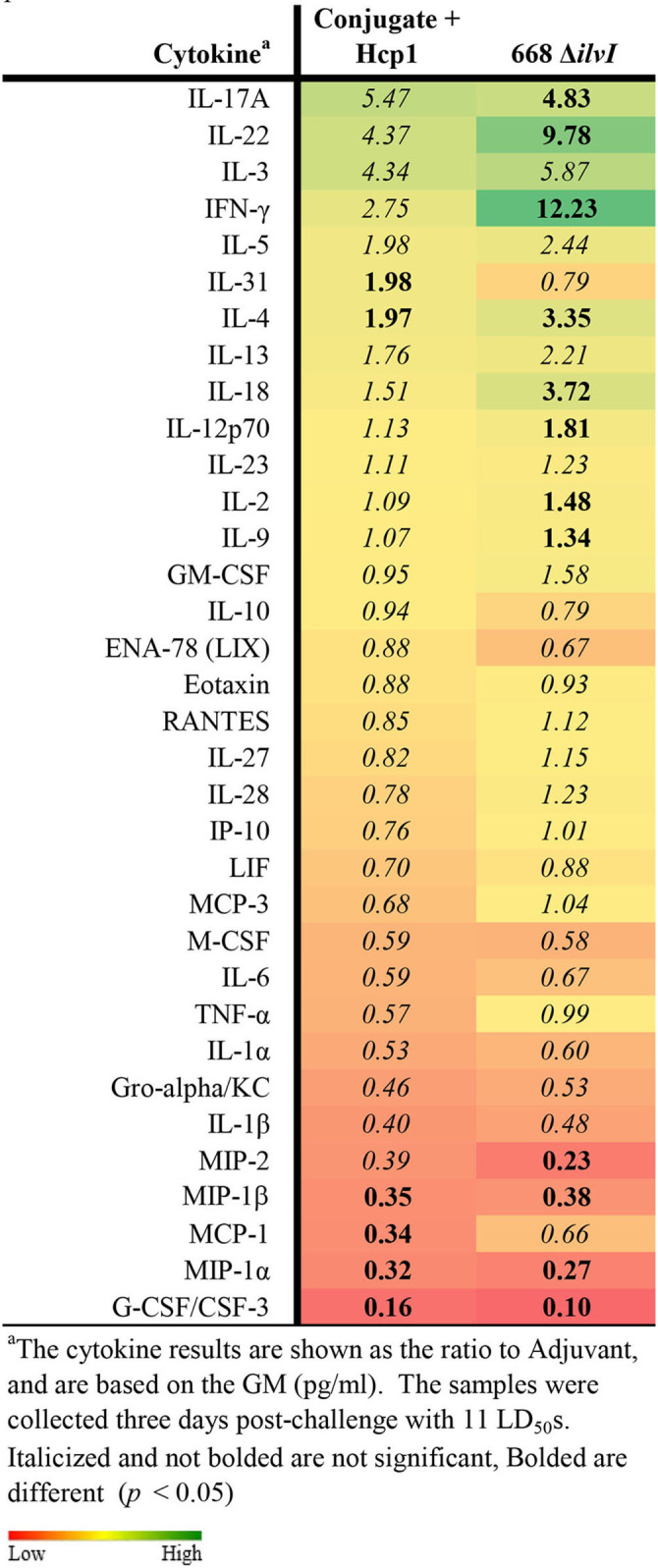
Fold change on day 3 post-challenge relative to adjuvant group of lung homogenates from vaccinated mice challenged with *Bp* MSHR5855.

The cytokine profiles of lung and spleen homogenates at three days post-challenge with *Bp* MSHR5855 were similar to the profiles generated following the *Bm* FMH challenge. Interestingly, contrary to the *Bm* FMH challenge, the IFN-γ, IL-4, IL-18, and IL-22 cytokine responses were greater in the 668 Δ*ilvI* vaccine group relative to the Conjugate + Hcp1 vaccine group after the *Bp* MSHR5855 challenge (Table 9, Supplementary Table 7); the presence of IL-22 in vaccinated mice may feasibly promote recovery post-challenge (Alcorn, 2020).

## Discussion

### Vaccine candidates for the pathogenic *Burkholderia*

A variety of defined proteins and polysaccharides, alone or as conjugates, that elicit varying degrees of protection have been previously assessed as potential vaccine components. These are exemplified by T3SS proteins (such as Bip), T6SS proteins (such as Hcp1), proteases, outer membrane proteins (OMPs, LolC), CPS, and LPS or purified OPS (Harland et al., 2007; Chin et al., 2012; Scott et al., 2014a,b, 2016; Gregory et al., 2015; Casey et al., 2016; Burtnick et al., 2018; Tomas-Cortazar et al., 2021). Hcp1 is required for assembly of the secretion apparatus and for the export of the T6SS virulence proteins (Mougous et al., 2006; Pukatzki et al., 2007; Brunet et al., 2014; Lim et al., 2015). Hcp1 is immunogenic and serodiagnostic for infection; the protein and anti-Hcp1 antibodies are present in melioidosis patient sera (Burtnick et al., 2011; Lim et al., 2015; Pumpuang et al., 2017; Phokrai et al., 2018); and Hcp1 is a putative protective antigen.

The surface-associated polysaccharide antigens, CPS and LPS, have been shown to be effective vaccine antigens (Nelson et al., 2004; Silva and Dow, 2013; Titball et al., 2017; Wang et al., 2020). They were shown to provide partial protection against lethal infection by parenteral routes (but not inhalational route) of exposure. However, although both antigens decreased the bacterial burden of infected animals, they failed to produce sterilizing immunity and failed to prevent chronic infection (Silva and Dow, 2013; Wang et al., 2020). Polysaccharides are known to be poor immunogens due to a lack of T cell involvement and an inability to stimulate a memory response and antibody class switching (Ishioka et al., 1992; Snapper, 2016). It has been shown that CPS and OPS immunogenicity can be increased by conjugation to carrier proteins such as tetanus toxoid protein fragment, flagellin proteins, BSA, Hcp1, or the CRM197 protein, as described above (Burtnick et al., 2012a,b, 2018; Scott et al., 2014a,b, 2016; Gregory et al., 2015; Titball et al., 2017). The conjugation of CPS to the highly immunogenic CRM197 protein carrier was protective in mice; complete protection was achieved (35 days post-challenge) by the inclusion of Hcp1; and remarkably, organs from the majority of the vaccinated mice lacked culturable bacteria (Burtnick et al., 2018). To potentially enhance efficacy of the capsule and Hcp1 combination, AhpC was added to the subunit combination in the current investigation (Burtnick et al., 2018). AhpC was strongly immunogenic for T cells from *Bp*-seropositive humans across diverse HLA types, and it stimulated strong T cell immunity in humanized HLA transgenic mice and humans (Reynolds et al., 2015; Dunachie et al., 2017). Patients with acute melioidosis who survived infection exhibited stronger T cell responses to AhpC compared to non-survivors (Reynolds et al., 2015; Dunachie et al., 2017). Although its effects on augmenting the Conjugate + Hcp1 vaccine immune response in the current effort were minor, Schmidt et al. have further characterized AhpC as a novel vaccine candidate for melioidosis (Schmidt et al., 2022).

Numerous LAV strains have been shown to be safe and protective against melioidosis and glanders; however, sterile immunity was not achieved reproducibly. The attenuation of virulence was achieved by mutating genes encoding factors required for virulence (e.g., *hcp1* and the *wcb* operon) or for biosynthesis of compounds essential for growth (e.g., amino acids) and unavailable in the host, i.e., *aroC, purM, asd, hisF, ilvI*, and *tonB* (Srilunchang et al., 2009; Warawa et al., 2009; Norris et al., 2011; Silva et al., 2013; Hatcher et al., 2016; Amemiya et al., 2019; Khakhum et al., 2019).

Several findings supported the selection of 668 Δ*ilvI* as a prototype live vaccine for melioidosis. Atkins et al. characterized *Bp* strain 2D2, which was also deficient in branched chain amino acid syntheses *(ilvI*) and highly attenuated in mice (Atkins et al., 2002). That strain was produced by transposon insertion mutagenesis of wild type *Bp* strain 576, which has a type B LPS. Alternately, the 668 Δ*ilvI* mutant has a complete deletion mutation of *ilvI* in strain MSHR668, which has a type A LPS. Type A LPS strains are far more prevalent than type B LPS strains.

### Comparison of protective efficacy and immune responses of the vaccine candidates

#### Protective efficacy

Our current and previous studies showed that the experimental Conjugate + Hcp1 (+/–AhpC) vaccines or the strain MSHR668-derived 668 Δ*ilvI* vaccine candidate afforded significant protection against aerosol challenge with the challenge strain *Bp* K96243. The vaccines were somewhat less protective against challenge with *Bp* MSHR5855 despite exposure to similar inhaled LD_50_ equivalents. In contrast, both vaccines produced strong protection against glanders. The 668 Δ*ilvI*-vaccinated mice had a more extended TTM in the early phase of the glanders infection compared to the Conjugate + Hcp1 vaccinees. However, despite the chronic and extended course often observed for glanders and melioidosis, most *Burkholderia* vaccine studies only monitor for protection for up to 1 month after challenge. The unusually long post-challenge monitoring period of the current studies emphasized the durability of the vaccine protection, especially against *Bm*.

We routinely perform inhalational challenges using a whole body-aerosol system, whereas some other studies have been performed using nose-only exposure systems. While the whole-body aerosol exposure system offers several logistical advantages compared to nose-only systems (e.g., causing less stress on the animals during exposure and allowing for the exposure of more animals simultaneously in order to minimize variability), it is important to note this difference between our study and other published data (Stephenson et al., 1988; Yeh et al., 1990; Oyabu et al., 2016). Differences in bacterial delivery strategies are also potential topics of future work, particularly in context of aerosolized *B. pseudomallei* and the diversity of clinical presentations that appear several months after exposure and cessation of the acute phase of disease.

#### Humoral immune responses

Using sera collected 28 days post-final vaccination, all vaccines elicited differing but significantly elevated titers to complex whole-cell killed antigens (BpK and Bm FMH), but only the Conjugate + Hcp1 vaccines produced a significant antibody response to the Hcp1 and CPS antigens (and to the AhpC antigen in mice vaccinated with it). Protection of the subunit-vaccinated mice was associated with the greatly increased levels of anti-Hcp1 and anti-CPS antibodies compared to the other groups, whereas protection of 668 Δ*ilvI*-vaccinated mice was associated with elevated titers to BpK and OPS and not to the three defined antigens (Hcp1, AhpC, and CPS). The reduced responses by the 668 Δ*ilvI* group to inactivated Bm FMH compared to that of BpK suggest that there are differences in the surface antigen profiles of *Bp* and *Bm*. The humoral responses assessed at an earlier time-point, i.e., 6 days after the last vaccination, largely reiterated those observed pre-challenge.

The higher IgG2c/IgG1 ratios to the killed BpK and Bm FMH antigens of the LAV group compared to the subunit group suggested that 668 Δ*ilvI* elicited a stronger Th1 response, possibly indicative of a greater role for cell-mediated immunity (Philipovskiy and Smiley, 2007). The exceedingly high anti-CPS IgG titers of the subunit vaccinated mice, and their lower IgG2c/IgG1 ratio of anti-CPS antibodies compared to that of the LAV, again implied that the subunit vaccines stimulated a more Th2 polarized humoral response as opposed to the more balanced or Th1 skewed responses elicited by LAV. However, the anti-Hcp1 response in the Conjugate + Hcp1 vaccinated mice appeared to be exceptionally Th1-polarized (high IgG2c/IgG1 ratio). Hence, the establishment and maintenance of discrete T cell responses is antigen dependent.

The vaccine literature supports an association with protection against lethal infection and the humoral antibody responses to certain defined and whole-cell antigens, and this association is dependent on vaccine type. The expression of Hcp1 during infection and its preferential antigen presenting cell binding activity reinforce both the role of Hcp1 alone in adaptive immunity against melioidosis, as shown here and previously, as well as its ability to significantly potentiate protection when given in combination with the CPS antigen (Burtnick et al., 2018). The protective capacity of anti-CPS antibodies alone has also been associated with protection. For example, vaccination of mice or hamsters with CPS or LPS induced antibody-dependent protection (Nelson et al., 2004; Silva and Dow, 2013; Scott et al., 2014a,b). Furthermore, passive transfer of immune sera, or monoclonal antibodies against these polysaccharides was also efficacious (Nelson et al., 2004; AuCoin et al., 2012; Silva and Dow, 2013). In our studies, the antibody titers to Hcp1 and CPS were associated with protection afforded by only the defined subunit vaccines, whereas 668 Δ*ilvI* elicited high IgG titers to the OPS. These data suggest that the CPS and LPS polysaccharides were not significantly cross-reactive. Whereas, the LAV generated a robust response against OPS, it did not induce a significant immune response to CPS (or Hcp1). The lack of anti-CPS antibodies raised against the LAV is not surprising and does not necessarily reflect the level of CPS expressed by the strain. Our interpretation of these data is that CPS is poorly immunogenic unless conjugated to a carrier protein. This is in contrast to the LPS which stimulates very robust responses against the O-antigen. The AhpC protein is a proven immunostimulant (O'Riordan et al., 2012; Guo et al., 2017). Moreover, the high IgG anti-AhpC titers detected previously in patients with acute melioidosis suggested that AhpC is immunogenic and expressed by *Bp in vivo* (Sengyee et al., 2021). However, when incorporated with Conjugate + Hcp1, the resulting anti-AhpC titers did not differ significantly between survivors and non-survivors, and the humoral and cell-mediated responses to this antigen were not clearly associated with enhanced protection, as detailed below.

In the *Bp* K96423 challenge study, humoral responses to whole-cell antigen BpK were more strongly associated with protection by the LAV than by the subunit vaccines (this association was not evident by the antibody responses to inactivated whole Bm FMH, as described above). Earlier studies also demonstrated that protection can be afforded by live or inactivated whole-cell *Burkholderia* vaccines (Whitlock et al., 2008; Sarkar-Tyson et al., 2009; Puangpetch et al., 2014; Wang et al., 2020). The studies showed that inactivated whole cells induced high antibody titers but these did not correlate directly with protection; it was hypothesized that other responses involving CD4+ T cells may also play a role in protection (Healey et al., 2005). Partial protection was elicited by heat- and formaldehyde-inactivated *Bp* and both induced high IgG titers to the vaccines (Puangpetch et al., 2014), but protection correlated most closely with pre-challenge levels of IFN-γ. In studies with a heat-killed *Bm* vaccine and mice depleted of B cells, TNF-α or IFN-γ, Whitlock showed that protection against lethal intraperitoneal infection by *Bm* required antibody and cell-mediated immune responses (Whitlock et al., 2008).

Khakhum et al. demonstrated a major role of the humoral immune responses in protection afforded by the Δ*tonB* Δ*hcp1* LAVs against lethal infections of C57BL/6 mice with *Bm* and *Bp* (Khakhum et al., 2019, 2020). These LAVs stimulated strong production of Th1 and Th17 cytokines, i.e., IFN-γ and IL-17A; however, T cell depletion studies showed that CD4+ and CD8+ T cells did not contribute significantly to vaccine-associated protection. Furthermore, in previous studies with a live attenuated *purM* mutant strain of *Bp*, survival was dependent on humoral immunity. It was shown that sera with high Ig titers passively transferred protection; mice lacking B cells were not protected by the live vaccine; and depletion of CD4+ or CD8+ T cells did not impact protection (Silva et al., 2013). Alternate findings were reported by others in studies conducted with live mutant auxotrophs of strain *Bp* MSHR668 in work performed in the sensitive BALB/c mouse model of melioidosis. As observed in the present report, among several mutants tested, the 668 Δ*ilvI* strains was among the most efficacious after high dose intraperitoneal infection. In contrast to the above studies, IgG class and subclass antibody responses to these mutants did not correlate with the amount of protection they provided. Splenocytes from mice vaccinated with 668 Δ*ilvI* expressed higher amounts of IFN-γ after stimulation with *Bp* antigens than cells from mice vaccinated with less protective candidates (Amemiya et al., 2019).

#### Cellular immune responses

Infection with the pathogenic *Burkholderia* and prophylactic vaccination are both known to stimulate cellular immune responses (Silva and Dow, 2013), and in the current study, these responses were evaluated using isolated splenocytes and organ homogenates. Splenocytes collected pre-challenge with *Bp* K96243 were stimulated with the Hcp1 and AhpC antigens to assess changes in cytokine responses. Compared to the Adjuvant controls, Hcp1 stimulation of the day 44 and 65 splenocytes upregulated numerous cytokines, most prominently in the Conjugate + Hcp1 group, whereas cytokine production was generally less stimulated or suppressed with 668 Δ*ilvI* splenocytes. There was a notable decrease overall in the extent of cytokine production in the day 65 splenocytes compared to day 44, yet the levels of a majority of the cytokines tested in day 65 samples were upregulated by Hcp1 in the Conjugate + Hcp1 group relative to the other groups (especially 668 Δ*ilvI*), e.g., MCP-3, IL-17A, and IFN-γ and many others (Supplementary Table 1). These and others were down-regulated in the 668 Δ*ilvI* group, with the exception of the pronounced increase in IL-17A and IL-31. IFN-γ is the major Th1 cytokine and is considered to be a correlate of immunity to infection with *Bp* (Santanirand et al., 1999; Ketheesan et al., 2002; Tippayawat et al., 2009; Silva and Dow, 2013; Jenjaroen et al., 2015; Dunachie et al., 2017; Burtnick et al., 2018; Sengyee et al., 2021). After infection, IFN-γ, mainly produced by NK cells and CD4^+^ T cells, stimulates macrophage activation, inducing high level of Th1 proinflammatory cytokines (e.g., IL-12, IL-2, IL-18, IL-27) and low levels of anti-inflammatory molecules (Spellberg and Edwards, 2001; Varma et al., 2002; Souza-Fonseca-Guimaraes et al., 2012; Kanevskiy et al., 2013). This promotes immune responses that enhance macrophage phagocytic ability to eliminate intracellular pathogens (Spellberg and Edwards, 2001; Mosser and Edwards, 2008; Murray and Wynn, 2011).

The low levels of IFN-γ secreting splenocytes after Hcp1 stimulation in the LAV group suggests that 668 Δ*ilvI* produced lower levels of surface/excreted Hcp1 compared to the subunit vaccine. A similar explanation may account for the markedly elevated level of MCP-3 from the Hcp1-stimulated cells of the Conjugate + Hcp1+/–AhpC mice, in contrast to the lack of MCP-3 stimulation in those of the 668 Δ*ilvI* group. However, IL-17A was highly expressed in both the Conjugate + Hcp1 subunit (days 44 and 65) and LAV groups (day 65). MCP-3 plays a role in enhancing monocyte and neutrophil recruitment (Mercer et al., 2014; Inaba et al., 2020). IL-17A and IL-22 are proinflammatory Th17 cell cytokines that synergize to promote neutrophil recruitment early after infection, and increase neutrophil bactericidal activity *via* enhanced production of defensins and calcium binding proteins, resulting in pathogen clearance (Valeri and Raffatellu, 2016). Finally, an exception to the reduced cytokine pattern of the 668 Δ*ilvI* group was observed for IL-31, which was significantly more highly expressed in pre-challenge cells after Hcp1-stimulation. IL-31 is a Th2-associated cytokine with many activities, such as inducing MCP-3 secretion and enhancing IL-17A effects on IL-6 and IL-8 regulation (Castellani et al., 2010). The functions of the upregulated cytokines, as well as the Th17 cell-associated antibacterial defenses, could be associated with the more restrained overall cytokine response after *Bp* K96243 challenge of the vaccinated compared to control mice, and the concomitantly increased subsequent protection against infection.

Cytokine expression in unstimulated spleen and lung homogenates before and after challenge was also evaluated in the *Bp* K96243 study. For those obtained 6 days post-vaccination, there was little or no enhanced expression of cytokines of the vaccine groups relative to the PBS controls. The levels of expression in the day 65 samples from the vaccinated mice were generally reduced compared to the Adjuvant control. However, the day 65 collections from the 668 Δ*ilvI* group revealed the uniquely increased expression of several cytokines. The IL-3 and IL-13 levels of the lung homogenates were especially enhanced in the Δ*ilvI* mice (Supplementary Table 2A). IL-3, a multifunctional T cell cytokine, is known to synergize with the Th-2 associated cytokines IL-4 and IL-13 to promote the alternative activation of mouse and human monocytes (Borriello et al., 2015). Excessive levels of IL-13, and the functionally related IL-4, are associated with increased allergic responses (Th2 inflammation and asthma) and resistance to parasites; the inclusion of inhibitory receptors of these Th2 molecules in vaccines has augmented immunity to infections such as HIV (Wijesundara et al., 2013; Ranasinghe et al., 2014; Bao and Reinhardt, 2015). However, IL-13 has also been associated with protection against murine bacterial sepsis and infections such as listeriosis (Flesch et al., 1997; Cao et al., 2012). The Conjugate + Hcp1 vaccine induces responses that more specifically target Hcp1 and CPS, and which are Th1 and Th2 polarized, respectively, as suggested also by the anti-Hcp1 and CPS antibody titers determined at the two time points. As expected, vaccination with 668 Δ*ilvI* generated a more diverse humoral response against BpK and OPS, and one that is more Th1/Th2-balanced. The cytokine response of the day 65 668 Δ*ilvI* lung homogenates revealed a pattern of expression that was more upregulated compared to that of the subunit groups, which may be attributed to prolonged inflammation and antigen persistence of the LAV relative to subunit vaccines.

Cytokine profiles of lung and spleen homogenates from mice were also evaluated 3 days post-challenge with *Bp* K96243. The three vaccine groups generally exhibited a significant reduction in cytokine levels compared to the controls. Exceptions were elevated IFN-γ and IL-22 cytokines in lung homogenates of the vaccinated mice, most significantly for those receiving 668 Δ*ilvI*. The perturbations in cytokine concentrations were much greater in the lung homogenates relative to spleen homogenates partly due to limited dissemination of *Bp* K96243 to the spleen at that time point. Aside from the anti-microbial effect of IFN-γ the immunoregulatory functions may also play a role since elevated IFN-γ levels in the vaccinated groups after *Bp* K96243 challenge (day 79) may limit excessive neutrophil infiltration into the lungs during the acute stages of infection, with neutrophilia in the lungs being potentially indicative of poor survival outcome due to suboptimal Th1 mediated immune response (Nandi and Behar, 2011; Bearss et al., 2017; Kanno et al., 2019). IL-22 enhances lung tissue integrity and reduces bacterial dissemination, specifically in the lungs; the increased expression of IL-22 in vaccinated animals, together with elevated IFN-γ, might serve as correlates of immunity. Upregulation of IL-22 may be a compensatory mechanism to mitigate further lung injury in 668 Δ*ilvI* vaccinated mice relative to subunit vaccinated groups with lower levels of inflammation based on day 65 lung cytokine levels (Pociask et al., 2013). These early post-challenge increases in IL-22 and IFN-γ cytokines might serve as correlates of immunity. They were associated with the greater extent of protection (rate and duration of survival) provided by both the Conjugate + Hcp1 vaccine and 668 Δ*ilvI*; and with the lack of significant survival advantage associated with AhpC (in these study conditions).

The cytokine responses post-challenge with *Bp* strain MSHR5855 of the Conjugate + Hcp1- and 668 Δ*ilvI*-vaccinated mice largely reiterated those post-K96243 challenge, i.e., the relative down-regulation of many cytokines compared to the controls, in contrast to the enhanced IL-22 and IFN-γ levels. Differences in responses after MSHR5855 challenge compared to those post-exposure to K96243 were observed, e.g., upregulated IL-17A and greater overall production of several Th1 and Th2 cytokines by both 668 Δ*ilvI* and Conjugate + Hcp1 mice. These differences were not clearly associated with differences in vaccine-mediated protection and might instead be predictive of reduced vaccine efficacy.

Lastly, the change in cytokine levels (spleen, lung) between the pre-challenge and day 3 post-challenge levels of K96243-infected mice was also significant. There was an overall suppression (or reduced upregulation) of cytokine production in vaccinated mice compared to control groups, suggesting that the vaccines protected the mice against the destructive effects of a cytokine storm (Tisoncik et al., 2012). This comparison underscored the upregulated post-challenge expression of IFN-γ and IL-22 in lung samples of vaccinated groups relative to the levels in the control groups. With the exception of IFN-γ, Conjugate + Hcp1 vaccine induced a higher overall cytokine response and protection against K96243 relative to the Conjugate + Hcp1 + AhpC and 668 Δ*ilvI* vaccine groups (Table 7). In addition, greater induction of IL-18 in turn leads to increased protective levels of IFN-γ while controlled IL-1β upregulation mitigates excessive IL-1β dependent neutrophil recruitment to the lungs and thereby limits lung injury (Ceballos-Olvera et al., 2011).

Cytokine profiles of lung and spleen homogenates from mice were also evaluated 3 days post-challenge with *Bm*. The responses were comparable to those observed after *Bp* K96243 and MSHR5855 challenges, i.e., a pattern of upregulation of a few cytokines such as IL-17A, IFN-γ, and IL-22, more so for the Conjugate + Hcp1 than the 668 Δ*ilvI* group in *Bm*-challenged mice, and with a larger number of cytokines being down-regulated compared to controls.

The 668 Δ*ilvI* and Conjugate + Hcp1 vaccines are intrinsically different prophylactics that appear to induce humoral and cellular immune responses which are qualitatively and quantitatively different, but similarly effective. Our findings add to the growing evidence for immune correlates of protection. In the current study, IFN-γ was highly induced by Hcp1 in splenocytes of the subunit groups at both the pre-challenge time points, as expected, and was highly expressed in unstimulated lung homogenates collected after, but not before, challenge. In contrast, IFN-γ was not induced in 668 Δ*ilvI* splenocytes by Hcp1, again suggesting a low expression of this antigen by the LAV *in vivo*. However, it was upregulated in the unstimulated pre-challenge (~2-fold), and especially post-challenge (~15-fold), homogenates from the 668 Δ*ilvI* vaccinees. These data confirm the role of IFN-γ and IL-22 in immunity to melioidosis and appear to suggest that the IFN-γ and IL-22 responses of lung homogenates align most closely with protection afforded with both the LAV and subunit vaccines. Much like with *Bp*, IFN-γ, and IL-22 also appear to play a protective role after *Bm* challenge in both the subunit and 668 Δ*ilvI* vaccinated mice.

### Vaccines and sterilizing immunity

We showed that by 3 days after challenge, the vaccinated animals cleared or reduced the infection quicker than the unvaccinated control animals. These data demonstrated an inverse correlation between mouse survival and bacterial load in the blood, spleens, and lungs on day 3 post-challenge. The bacterial burden in the lungs and spleens also appeared to correlate with the intensity of the cytokine response, as discussed below. The bacteriological results of mice surviving to day 60 were not as closely related to survival as were the 3 day post-challenge data with the three strains, largely due to the small number of positive cultures. Nevertheless, a majority of mouse survivors vaccinated with Conjugate + Hcp1 or 668 Δ*ilvI* had cleared infection with *Bp* K96243, or greatly reduced the number of CFU of *Bm* FMH or *Bp* MSHR5855, from all tissue types sampled. An important caveat to these results is the fact that we sampled only blood, lungs, and spleens. Due to the heterogeneous clinical presentation of these organisms in the mouse model, it remains a possibility that reservoirs of bacteria could be present in other tissues (despite lack of gross evidence upon necropsy) or that the bacteria may have entered a persistent or non-culturable state (Pumpuang et al., 2011; Li et al., 2014; Lewis and Torres, 2016).

### Conclusions

Past investigations suggested a compelling, though not universal, role for humoral immunity in vaccine-elicited protection in animal models of melioidosis (Nieves et al., 2014; Titball et al., 2017; Khakhum et al., 2020). Furthermore, *Bp*-specific antibody responses play a major role in protection from lethal disease of infected people. High titers of anti-*Bp* IgG2 in Thai patients with acute melioidosis were associated with survival (Chaichana et al., 2021). In other studies, Chenthamarakshan and coworkers corroborated the predominance of IgG1 and IgG2 antibodies specific for *Bp* in sera collected 1–3 weeks after onset of illness from Malaysian patients (Chenthamarakshan et al., 2001). High IgG3 titers to the Hcp1 and OPS antigens correlated with survival in Thai patients. These and similar human data will assist in guiding the selection of optimal *Burkholderia* vaccines.

Our results of the *Bp* strain K96243 challenge trial revealed an inverse association between excessive cytokine production and vaccine-induced protection against lethal infection; and the *Bm* FMH challenge study indirectly supported this. The generally more controlled release in mice of cytokines in vaccinated compared to controls after K96243 aerosol challenge suggested that a cytokine storm occurred in the PBS and Adjuvant control groups with elevated levels of IL-6, Gro-α/CXCL1, IL-18, and IL-1β. Increased IL-6 levels can lead to an acute phase response (inducing expression of C-reactive protein and fibrinogen, thereby enhancing localized infection) and is correlated with the severity of sepsis (Damas et al., 1992; Song et al., 2019). Gro-α/CXCL1 is a chemoattractant for neutrophils, IL-18 is a Th1 pro-inflammatory cytokine and IFN-γ inducer, and IL-1β is a pro-inflammatory cytokine (Dinarello, 1996, 2000; Sawant et al., 2016). Thus, the more controlled cytokine production in the 668 Δ*ilvI*- and Conjugate + Hcp1-vaccinated groups, together with enhanced production of cytokines suppressing bacterial dissemination and promoting lung epithelial integrity such as IL-22 and IL-17A (Valeri and Raffatellu, 2016; Alcorn, 2020), were correlated with protection by the attenuated mutant and subunit vaccines.

The cellular responses of patients with melioidosis have been reported, and the findings may inform vaccine development. For example, Krishnananthasivam et al. described differential gene expression of key cytokines involved in human host responses that can distinguish melioidosis cases from other infections and from healthy individuals. They revealed that dysregulated cytokine responses (upregulated Th2 and Th17 cytokine and a downregulated Th1 cytokine response) compared to healthy controls were predictive of disease severity, due to cytokine-associated damage. Such changes in cytokine levels were proposed to be potential diagnostic biomarkers for melioidosis and for monitoring disease progression (Krishnananthasivam et al., 2017). Others have characterized plasma levels and/or mRNA expression of pro-and anti-inflammatory cytokines in melioidosis patients for similar purposes. For instance, levels of IFN-γ, IL-6, IL-8, IL-10, IL-23, and TNF-α were reported to be higher in melioidosis cases compared with healthy individuals (Kaewarpai et al., 2020; Sengyee et al., 2021; Wright et al., 2021). All of them except IFN-γ were associated with 28-day mortality. IL-8 provided the best discrimination of mortality, and over time, non-survivors had increasing IL-6, IL-8, and IL-17A levels, in contrast to survivors (Kaewarpai et al., 2020; Wright et al., 2021). Similarly, Sengygee and coworkers correlated patient survival with high IFN-γ responses (Sengyee et al., 2021). Of interest, the latter group discovered that, although high IgG levels against Hcp1 and AhpC were present in sera from patients with acute melioidosis, there were no significant differences between survivors and non-survivors. Determining the vaccine-associated changes in cytokine responses (particularly in lungs) over time before and after virulent challenge and their correlation with protection, as initiated in the *Bp* K96243 study, will support vaccine selection. Additional work will need to examine alternate vaccine delivery mechanism to include mucosal delivery. It was previously shown that mucosal delivery may enhance protection in mouse models of melioidosis (Easton et al., 2011; Henderson et al., 2011; Rhee, 2020). These novel concepts will be critical for vaccine optimization and the determination of immune-correlates in the future. When taken together, our data support the development of both vaccine platforms. While subunit vaccines have obvious safety and stability advantages, live attenuated vaccines can be similarly protective and may require less doses for immunity. Lastly, heterologous vaccination protocols may be warranted to combat a disease that is considered refractory to a single vaccine approach and by combining the beneficial characteristics of a subunit vaccine and a live attenuated vaccine a more complete and potentially longer lasting immune response could be achievable.

## Data availability statement

The raw data supporting the conclusions of this article will be made available by the authors, without undue reservation.

## Ethics statement

The animal studies were reviewed and approved by United States Army Medical Research Institute of Infectious Diseases (USAMRIID) Institutional Animal Care and Use Committee (IACUC).

## Author contributions

SB, CC, MB, PB, and DD designed and supervised the project. SB, CC, CK, JD, NR, JS, MH, ZS, IV, ZH, RR-A, YT, LS, and CO prepared vaccine antigens and performed the experiments. SB, CC, CK, JD, JS, MH, DF, SW, and DD performed data analyses. SB, CC, and SW wrote the manuscript. SB, CC, MB, PB, SW, and DD edited manuscript. All authors contributed to the article and approved the submitted version.

## Funding

This work was funded by the U.S. Defense Threat Reduction Agency, project JSTO-CBD CB10207 (USAMRIID) and HDTRA1-18-C-0062 (University of Nevada, Reno).

## Conflict of interest

The authors declare that the research was conducted in the absence of any commercial or financial relationships that could be construed as a potential conflict of interest.

## Publisher's note

All claims expressed in this article are solely those of the authors and do not necessarily represent those of their affiliated organizations, or those of the publisher, the editors and the reviewers. Any product that may be evaluated in this article, or claim that may be made by its manufacturer, is not guaranteed or endorsed by the publisher.

## Author disclaimer

Opinions, interpretations, conclusions, and recommendations are those of the authors and are not necessarily endorsed by the U.S. Army.

## References

[B1] Alcorn J. F. (2020). IL-22 Plays a critical role in maintaining epithelial integrity during pulmonary infection. Front. Immunol. 11, 1160. 10.3389/fimmu.2020.01160 32582219 PMC7296169

[B2] Amemiya K. Dankmeyer J. L. Biryukov S. S. Trevino S. R. Klimko C. P. Mou S. M.. (2019). Deletion of two genes in *Burkholderia pseudomallei* MSHR668 that target essential amino acids protect acutely infected BALB/c mice and promote long term survival. Vaccines 7, 196. 10.3390/vaccines7040196 31779073 PMC6963190

[B3] Atkins T. Prior R. G. Mack K. Russell P. Nelson M. Oyston P. C.. (2002). A mutant of *Burkholderia pseudomallei*, auxotrophic in the branched chain amino acid biosynthetic pathway, is attenuated and protective in a murine model of melioidosis. Infect. Immun. 70, 5290–5294. 10.1128/IAI.70.9.5290-5294.2002 12183585 PMC128252

[B4] AuCoin D. P. Reed D. E. Marlenee N. L. Bowen R. A. Thorkildson P. Judy B. M.. (2012). Polysaccharide specific monoclonal antibodies provide passive protection against intranasal challenge with *Burkholderia pseudomallei*. PLoS ONE 7, e35386. 10.1371/journal.pone.0035386 22530013 PMC3328442

[B5] Baker S. M. Davitt C. J. H. Motyka N. Kikendall N. L. Russell-Lodrigue K. Roy C. J.. (2017). A *Burkholderia pseudomallei* outer membrane vesicle vaccine provides cross protection against inhalational glanders in mice and non-human primates. Vaccines 5, 49. 10.3390/vaccines5040049 29232837 PMC5748615

[B6] Baker S. M. Settles E. W. Davitt C. Gellings P. Kikendall N. Hoffmann J.. (2021). *Burkholderia pseudomallei* OMVs derived from infection mimicking conditions elicit similar protection to a live-attenuated vaccine. NPJ Vaccines 6, 18. 10.1038/s41541-021-00281-z 33514749 PMC7846723

[B7] Bao K. Reinhardt R. L. (2015). The differential expression of IL-4 and IL-13 and its impact on type-2 immunity. Cytokine 75, 25–37. 10.1016/j.cyto.2015.05.008 26073683 PMC5118948

[B8] Bearss J. J. Hunter M. Dankmeyer J. L. Fritts K. A. Klimko C. P. Weaver C. H.. (2017). Characterization of pathogenesis of and immune response to *Burkholderia pseudomallei* K96243 using both inhalational and intraperitoneal infection models in BALB/c and C57BL/6 mice. PLoS ONE 12, e0172627. 10.1371/journal.pone.0172627 28235018 PMC5325312

[B9] Borriello F. Longo M. Spinelli R. Pecoraro A. Granata F. Staiano R. I.. (2015). IL-3 synergises with basophil-derived IL-4 and IL-13 to promote the alternative activation of human monocytes. Eur. J. Immunol. 45, 2042–2051. 10.1002/eji.201445303 25824485 PMC4496336

[B10] Bozue J. A. Chaudhury S. Amemiya K. Chua J. Cote C. K. Toothman R. G.. (2016). Phenotypic characterization of a novel virulence-factor deletion strain of *Burkholderia mallei* that provides partial protection against inhalational glanders in mice. Front. Cell Infect. Microbiol. 6, 21. 10.3389/fcimb.2016.00021 26955620 PMC4767903

[B11] Brunet Y. R. Henin J. Celia H. Cascales E. (2014). Type VI secretion and bacteriophage tail tubes share a common assembly pathway. EMBO Rep. 15, 315–321. 10.1002/embr.201337936 24488256 PMC3989698

[B12] Burtnick M. N. Brett P. J. Harding S. V. Ngugi S. A. Ribot W. J. Chantratita N.. (2011). The cluster 1 type VI secretion system is a major virulence determinant in *Burkholderia pseudomallei*. Infect. Immun. 79, 1512–1525. 10.1128/IAI.01218-10 21300775 PMC3067527

[B13] Burtnick M. N. Heiss C. Roberts R. A. Schweizer H. P. Azadi P. Brett P. J.. (2012a). Development of capsular polysaccharide-based glycoconjugates for immunization against melioidosis and glanders. Front. Cell Infect. Microbiol. 2, 108. 10.3389/fcimb.2012.00108 22912938 PMC3419357

[B14] Burtnick M. N. Heiss C. Schuler A. M. Azadi P. Brett P. J. (2012b). Development of novel O-polysaccharide based glycoconjugates for immunization against glanders. Front. Cell Infect. Microbiol. 2, 148. 10.3389/fcimb.2012.00148 23205347 PMC3506924

[B15] Burtnick M. N. Shaffer T. L. Ross B. N. Muruato L. A. Sbrana E. DeShazer D.. (2018). Development of subunit vaccines that provide high-level protection and sterilizing immunity against acute inhalational melioidosis. Infect. Immun. 86, e00724–17. 10.1128/IAI.00724-17 29109172 PMC5736816

[B16] Cao Y. Z. Tu Y. Y. Chen X. Wang B. L. Zhong Y. X. Liu M. H.. (2012). Protective effect of Ulinastatin against murine models of sepsis: inhibition of TNF-alpha and IL-6 and augmentation of IL-10 and IL-13. Exp. Toxicol. Pathol. 64, 543–547. 10.1016/j.etp.2010.11.011 21159497

[B17] Casey W. T. Spink N. Cia F. Collins C. Romano M. Berisio R.. (2016). Identification of an OmpW homologue in *Burkholderia pseudomallei*, a protective vaccine antigen against melioidosis. Vaccine 34, 2616–2621. 10.1016/j.vaccine.2016.03.088 27091689

[B18] Castellani M. L. Felaco P. Galzio R. J. Tripodi D. Toniato E. De Lutiis M. A.. (2010). IL-31 a Th2 cytokine involved in immunity and inflammation. Int. J. Immunopathol. Pharmacol. 23, 709–713. 10.1177/039463201002300304 20943040

[B19] Ceballos-Olvera I. Sahoo M. Miller M. A. Del Barrio L. Re F. (2011). Inflammasome-dependent pyroptosis and IL-18 protect against *Burkholderia pseudomallei* lung infection while IL-1β is deleterious. PLoS Pathog. 7, e1002452. 10.1371/journal.ppat.1002452 22241982 PMC3248555

[B20] Chaichana P. Jenjaroen K. Chumseng S. Sumonwiriya M. Rongkard P. Kronsteiner B.. (2021). Role of *Burkholderia pseudomallei*-specific IgG2 in adults with acute melioidosis, Thailand. Emerg. Infect. Dis. 27, 463–470. 10.3201/eid2702.200213 33496230 PMC7853568

[B21] Cheng A. C. Dance D. A. Currie B. J. (2005). Bioterrorism, glanders and melioidosis. Euro. Surveill. 10, E1–2; author reply E1-2. 10.2807/esm.10.03.00528-en 15827368

[B22] Chenthamarakshan V. Kumutha M. V. Vadivelu J. Puthucheary S. D. (2001). Distribution of immunoglobulin classes and IgG subclasses against a culture filtrate antigen of *Burkholderia pseudomallei* in melioidosis patients. J. Med. Microbiol. 50, 55–61. 10.1099/0022-1317-50-1-55 11192506

[B23] Chin C. Y. Tan S. C. Nathan S. (2012). Immunogenic recombinant *Burkholderia pseudomallei* MprA serine protease elicits protective immunity in mice. Front. Cell Infect. Microbiol. 2, 85. 10.3389/fcimb.2012.00085 22919676 PMC3417646

[B24] Cote C. K. Biryukov S. S. Klimko C. P. Shoe J. L. Hunter M. Rosario-Acevedo R.. (2021). Protection elicited by attenuated live yersinia pestis vaccine strains against lethal infection with virulent *Y. pestis*. Vaccines 9, 16. 10.3390/vaccines9020161 33669472 PMC7920443

[B25] Currie B. J. (2015). Melioidosis: evolving concepts in epidemiology, pathogenesis, and treatment. Semin. Respir. Crit. Care Med. 36, 111–125. 10.1055/s-0034-1398389 25643275

[B26] Damas P. Ledoux D. Nys M. Vrindts Y. De Groote D. Franchimont P.. (1992). Cytokine serum level during severe sepsis in human IL-6 as a marker of severity. Ann. Surg. 215, 356–362. 10.1097/00000658-199204000-00009 1558416 PMC1242452

[B27] Dance D. A. Limmathurotsakul D. (2018). Global burden and challenges of melioidosis. Trop. Med. Infect. Dis. 3, 13. 10.3390/tropicalmed3010013 30274411 PMC6136634

[B28] Dinarello C. A. (1996). Biologic basis for interleukin-1 in disease. Blood 87, 2095–2147. 10.1182/blood.V87.6.2095.bloodjournal8762095 8630372

[B29] Dinarello C. A. (2000). Interleukin-18, a proinflammatory cytokine. Eur. Cytokine Netw. 11, 483–486. 11203186

[B30] Dunachie S. J. Jenjaroen K. Reynolds C. J. Quigley K. J. Sergeant R. Sumonwiriya M.. (2017). Infection with *Burkholderia pseudomallei*- immune correlates of survival in acute melioidosis. Sci. Rep. 7, 12143. 10.1038/s41598-017-12331-5 28939855 PMC5610189

[B31] Easton A. Haque A. Chu K. Patel N. Lukaszewski R. A. Krieg A. M.. (2011). Combining vaccination and postexposure CpG therapy provides optimal protection against lethal sepsis in a biodefense model of human melioidosis. J. Infect. Dis. 204, 636–644. 10.1093/infdis/jir301 21791666 PMC3144166

[B32] Felgner P. L. Kayala M. A. Vigil A. Burk C. Nakajima-Sasaki R. Pablo J.. (2009). A *Burkholderia pseudomallei* protein microarray reveals serodiagnostic and cross-reactive antigens. Proc. Natl. Acad. Sci. U.S.A. 106, 13499–13504. 10.1073/pnas.0812080106 19666533 PMC2717108

[B33] Flesch I. E. Wandersee A. Kaufmann S. H. (1997). Effects of IL-13 on murine listeriosis. Int. Immunol. 9, 467–474. 10.1093/intimm/9.4.467 9138006

[B34] Fuller T. E. Thacker B. J. Duran C. O. Mulks M. H. (2000). A genetically-defined riboflavin auxotroph of *Actinobacillus pleuropneumoniae* as a live attenuated vaccine. Vaccine 18, 2867–2877. 10.1016/S0264-410X(00)00076-1 10812230

[B35] Galyov E. E. Brett P. J. DeShazer D. (2010). Molecular insights into *Burkholderia pseudomallei* and *Burkholderia mallei* pathogenesis. Annu. Rev. Microbiol. 64, 495–517. 10.1146/annurev.micro.112408.134030 20528691

[B36] Gregory A. E. Judy B. M. Qazi O. Blumentritt C. A. Brown K. A. Shaw A. M.. (2015). A gold nanoparticle-linked glycoconjugate vaccine against *Burkholderia mallei*. Nanomedicine 11, 447–456. 10.1016/j.nano.2014.08.005 25194998 PMC4330121

[B37] Guo S. H. Wang H. F. Nian Z. G. Wang Y. D. Zeng Q. Y. Zhang G.. (2017). Immunization with alkyl hydroperoxide reductase subunit C reduces *Fusobacterium nucleatum* load in the intestinal tract. Sci. Rep. 7, 10566. 10.1038/s41598-017-11127-x 28874771 PMC5585165

[B38] Harland D. N. Chu K. Haque A. Nelson M. Walker N. J. Sarkar-Tyson M.. (2007). Identification of a LolC homologue in *Burkholderia pseudomallei*, a novel protective antigen for melioidosis. Infect. Immun. 75, 4173–4180. 10.1128/IAI.00404-07 17517877 PMC1951986

[B39] Hatcher C. L. Mott T. M. Muruato L. A. Sbrana E. Torres A. G. (2016). *Burkholderia mallei* CLH001 attenuated vaccine strain is immunogenic and protects against acute respiratory glanders. Infect. Immun. 84, 2345–2354. 10.1128/IAI.00328-16 27271739 PMC4962637

[B40] Hayden H. S. Lim R. Brittnacher M. J. Sims E. H. Ramage E. R. Fong C.. (2012). Evolution of *Burkholderia pseudomallei* in recurrent melioidosis. PLoS ONE 7, e36507. 10.1371/journal.pone.0036507 22615773 PMC3352902

[B41] Healey G. D. Elvin S. J. Morton M. Williamson E. D. (2005). Humoral and cell-mediated adaptive immune responses are required for protection against *Burkholderia pseudomallei* challenge and bacterial clearance postinfection. Infect. Immun. 73, 5945–5951. 10.1128/IAI.73.9.5945-5951.2005 16113315 PMC1231116

[B42] Henderson A. Propst K. Kedl R. Dow S. (2011). Mucosal immunization with liposome-nucleic acid adjuvants generates effective humoral and cellular immunity. Vaccine 29, 5304–5312. 10.1016/j.vaccine.2011.05.009 21600950 PMC3539814

[B43] Houghton R. L. Reed D. E. Hubbard M. A. Dillon M. J. Chen H. Currie B. J.. (2014). Development of a prototype lateral flow immunoassay (LFI) for the rapid diagnosis of melioidosis. PLoS Negl. Trop. Dis. 8, e2727. 10.1371/journal.pntd.0002727 24651568 PMC3961207

[B44] Inaba A. Tuong Z. K. Riding A. M. Mathews R. J. Martin J. L. Saeb-Parsy K.. (2020). B Lymphocyte-derived CCL7 augments neutrophil and monocyte recruitment, exacerbating acute kidney injury. J. Immunol. 205, 1376–1384. 10.4049/jimmunol.2000454 32737150 PMC7444279

[B45] Ishioka G. Y. Lamont A. G. Thomson D. Bulbow N. Gaeta F. C. Sette A.. (1992). MHC interaction and T cell recognition of carbohydrates and glycopeptides. J. Immunol. 148, 2446–2451. 1560200

[B46] Jenjaroen K. Chumseng S. Sumonwiriya M. Ariyaprasert P. Chantratita N. Sunyakumthorn P.. (2015). T-Cell responses are associated with survival in acute melioidosis patients. PLoS Negl. Trop. Dis. 9, e0004152. 10.1371/journal.pntd.0004152 26495852 PMC4619742

[B47] Johansen P. Storni T. Rettig L. Qiu Z. Der-Sarkissian A. Smith K. A.. (2008). Antigen kinetics determines immune reactivity. Proc. Natl. Acad. Sci. U.S.A. 105, 5189–5194. 10.1073/pnas.0706296105 18362362 PMC2278203

[B48] Johnson M. M. Ainslie K. M. (2017). Vaccines for the prevention of melioidosis and glanders. Curr. Trop. Med. Rep. 4, 136–145. 10.1007/s40475-017-0121-7 29242769 PMC5724791

[B49] Kaewarpai T. Ekchariyawat P. Phunpang R. Wright S. W. Dulsuk A. Moonmueangsan B.. (2020). Longitudinal profiling of plasma cytokines in melioidosis and their association with mortality: a prospective cohort study. Clin. Microbiol. Infect. 26, 783.e1–783.e8. 10.1016/j.cmi.2019.10.032 31705997 PMC7647866

[B50] Kanevskiy L. M. Telford W. G. Sapozhnikov A. M. Kovalenko E. I. (2013). Lipopolysaccharide induces IFN-γ production in human NK cells. Front. Immunol. 4, 11. 10.3389/fimmu.2013.00011 23372571 PMC3556587

[B51] Kanno E. Tanno H. Masaki A. Sasaki A. Sato N. Goto M.. (2019). Defect of interferon γ leads to impaired wound healing through prolonged neutrophilic inflammatory response and enhanced MMP-2 activation. Int. J. Mol. Sci. 20, 5657. 10.3390/ijms20225657 31726690 PMC6888635

[B52] Ketheesan N. Barnes J. L. Ulett G. C. VanGessel H. J. Norton R. E. Hirst R. G.. (2002). Demonstration of a cell-mediated immune response in melioidosis. J. Infect. Dis. 18, 286–289. 10.1086/341222 12134268

[B53] Khakhum N. Bharaj P. Myers J. N. Tapia D. Kilgore P. B. Ross B. N.. (2019). *Burkholderia pseudomallei* Δ*tonB* Δ*hcp*1 live attenuated vaccine strain elicits full protective immunity against aerosolized melioidosis infection. mSphere 4, e00570–18. 10.1128/mSphere.00570-18 30602524 PMC6315081

[B54] Khakhum N. Bharaj P. Walker D. H. Torres A. G. Endsley J. J. (2021). Antigen-specific antibody and polyfunctional T cells generated by respiratory immunization with protective Burkholderia Δ*tonB* Δ*hcp*1 live attenuated vaccines. NPJ Vaccines 6, 72. 10.1038/s41541-021-00333-4 33986290 PMC8119421

[B55] Khakhum N. Chapartegui-Gonzalez I. Torres A. G. (2020). Combating the great mimicker: latest progress in the development of *Burkholderia pseudomallei* vaccines. Exp. Rev. Vaccines 19, 653–660. 10.1080/14760584.2020.1791089 32669008 PMC8062048

[B56] Krishnananthasivam S. Sathkumara H. D. Corea E. Natesan M. De Silva A. D. (2017). Gene expression profile of human cytokines in response to *Burkholderia pseudomallei* infection. mSphere 2, e00121–17. 10.1128/mSphere.00121-17 28435890 PMC5397567

[B57] Lewis E. R. Torres A. G. (2016). The art of persistence-the secrets to Burkholderia chronic infections. Pathog Dis. 74, ftw070. 10.1093/femspd/ftw070 27440810 PMC5985510

[B58] Li L. Mendis N. Trigui H. Oliver J. D. Faucher S. P. (2014). The importance of the viable but non-culturable state in human bacterial pathogens. Front. Microbiol. 5, 258. 10.3389/fmicb.2014.00258 24917854 PMC4040921

[B59] Lim Y. T. Jobichen C. Wong J. Limmathurotsakul D. Li S. Chen Y.. (2015). Extended loop region of Hcp1 is critical for the assembly and function of type VI secretion system in *Burkholderia pseudomallei*. Sci. Rep. 5, 8235. 10.1038/srep08235 25648885 PMC4650826

[B60] Limmathurotsakul D. Funnell S. G. Torres A. G. Morici L. A. Brett P. J. Dunachie S.. (2015). Consensus on the development of vaccines against naturally acquired melioidosis. Emerg. Infect. Dis. 21, e141480. 10.3201/eid2106.141480 25992835 PMC4451926

[B61] Limmathurotsakul D. Golding N. Dance D. A. Messina J. P. Pigott D. M. Moyes C. L.. (2016). Predicted global distribution of *Burkholderia pseudomallei* and burden of melioidosis. Nat. Microbiol. 1, 15008. 10.1038/nmicrobiol.2015.8 27571754

[B62] Marchetti R. Dillon M. J. Burtnick M. N. Hubbard M. A. Kenfack M. T. Bleriot Y.. (2015). *Burkholderia pseudomallei* capsular polysaccharide recognition by a monoclonal antibody reveals key details toward a biodefense vaccine and diagnostics against melioidosis. ACS Chem. Biol. 10, 2295–2302. 10.1021/acschembio.5b00502 26198038

[B63] Martin R. M. Brady J. L. Lew A. M. (1998). The need for IgG2c specific antiserum when isotyping antibodies from C57BL/6 and NOD mice. J. Immunol. Methods. 212, 187–192. 10.1016/S0022-1759(98)00015-5 9672206

[B64] Mercer P. F. Williams A. E. Scotton C. J. Jose R. J. Sulikowski M. Moffatt J. D.. (2014). Proteinase-activated receptor-1, CCL2, and CCL7 regulate acute neutrophilic lung inflammation. Am. J. Respir. Cell Mol. Biol. 50, 144–157. 10.1165/rcmb.2013-0142OC 23972264 PMC3930934

[B65] Mosser D. M. Edwards J. P. (2008). Exploring the full spectrum of macrophage activation. Nat. Rev. Immunol. 8, 958–969. 10.1038/nri2448 19029990 PMC2724991

[B66] Mougous J. D. Cuff M. E. Raunser S. Shen A. Zhou M. Gifford C. A.. (2006). A virulence locus of *Pseudomonas aeruginosa* encodes a protein secretion apparatus. Science 312, 1526–1530. 10.1126/science.1128393 16763151 PMC2800167

[B67] Murray P. J. Wynn T. A. (2011). Protective and pathogenic functions of macrophage subsets. Nat. Rev. Immunol. 11, 723–737. 10.1038/nri3073 21997792 PMC3422549

[B68] Nandi B. Behar S. M. (2011). Regulation of neutrophils by interferon-γ limits lung inflammation during tuberculosis infection. J. Exp. Med. 208, 2251–2262. 10.1084/jem.20110919 21967766 PMC3201199

[B69] National Research Council (2011). Guide for the Care and Use of Laboratory Animals: Eighth Edition. Washington, DC: The National Academies Press.

[B70] Nelson M. Prior J. L. Lever M. S. Jones H. E. Atkins T. P. Titball R. W.. (2004). Evaluation of lipopolysaccharide and capsular polysaccharide as subunit vaccines against experimental melioidosis. J. Med. Microbiol. 53, 1177–1182. 10.1099/jmm.0.45766-0 15585494

[B71] Nieves W. Asakrah S. Qazi O. Brown K. A. Kurtz J. Aucoin D. P.. (2011). A naturally derived outer-membrane vesicle vaccine protects against lethal pulmonary *Burkholderia pseudomallei* infection. Vaccine 29, 8381–8389. 10.1016/j.vaccine.2011.08.058 21871517 PMC3195868

[B72] Nieves W. Petersen H. Judy B. M. Blumentritt C. A. Russell-Lodrigue K. Roy C. J.. (2014). A *Burkholderia pseudomallei* outer membrane vesicle vaccine provides protection against lethal sepsis. Clin. Vaccine Immunol. 21, 747–754. 10.1128/CVI.00119-14 24671550 PMC4018892

[B73] Norris M. H. Propst K. L. Kang Y. Dow S. W. Schweizer H. P. Hoang T. T.. (2011). The *Burkholderia pseudomallei* Δasd mutant exhibits attenuated intracellular infectivity and imparts protection against acute inhalation melioidosis in mice. Infect. Immun. 79, 4010–4018. 10.1128/IAI.05044-11 21807903 PMC3187240

[B74] O'Callaghan D. Maskell D. Liew F. Y. Easmon C. S. Dougan G. (1988). Characterization of aromatic- and purine-dependent *Salmonella typhimurium*: attention, persistence, and ability to induce protective immunity in BALB/c mice. Infect. Immun. 56, 419–423. 10.1128/iai.56.2.419-423.1988 3276625 PMC259298

[B75] O'Riordan A. A. Morales V. A. Mulligan L. Faheem N. Windle H. J. Kelleher D. P.. (2012). Alkyl hydroperoxide reductase: a candidate *Helicobacter pylori* vaccine. Vaccine 30, 3876–3884. 10.1016/j.vaccine.2012.04.002 22512976

[B76] Oyabu T. Morimoto Y. Izumi H. Yoshiura Y. Tomonaga T. Lee B. W.. (2016). Comparison between whole-body inhalation and nose-only inhalation on the deposition and health effects of nanoparticles. Environ. Health Prev. Med. 21, 42–48. 10.1007/s12199-015-0493-z 26438563 PMC4693768

[B77] Parthasarathy N. DeShazer D. England M. Waag D. M. (2006). Polysaccharide microarray technology for the detection of *Burkholderia pseudomallei* and *Burkholderia mallei* antibodies. Diagn. Microbiol. Infect. Dis. 56, 329–332. 10.1016/j.diagmicrobio.2006.04.018 16765554 PMC7127370

[B78] Peacock S. J. Limmathurotsakul D. Lubell Y. Koh G. C. White L. J. Day N. P.. (2012). Melioidosis vaccines: a systematic review and appraisal of the potential to exploit biodefense vaccines for public health purposes. PLoS Negl. Trop. Dis. 6, e1488. 10.1371/journal.pntd.0001488 22303489 PMC3269417

[B79] Philipovskiy A. V. Smiley S. T. (2007). Vaccination with live *Yersinia pestis* primes CD4 and CD8 T cells that synergistically protect against lethal pulmonary *Y. pestis* infection. Infect. Immun. 75, 878–885. 10.1128/IAI.01529-06 17118978 PMC1828512

[B80] Phokrai P. Karoonboonyanan W. Thanapattarapairoj N. Promkong C. Dulsuk A. Koosakulnirand S.. (2018). A rapid immunochromatography test based on Hcp1 is a potential point-of-care test for serological diagnosis of melioidosis. J. Clin. Microbiol. 56, e00346–18. 10.1128/JCM.00346-18 29848565 PMC6062804

[B81] Pociask D. A. Scheller E. V. Mandalapu S. McHugh K. J. Enelow R. I. Fattman C. L.. (2013). IL-22 is essential for lung epithelial repair following influenza infection. Am. J. Pathol. 182, 1286–1296. 10.1016/j.ajpath.2012.12.007 23490254 PMC3620404

[B82] Puangpetch A. Anderson R. Huang Y. Y. Saengsot R. Sermswan R. W. Wongratanacheewin S.. (2014). Comparison of the protective effects of killed *Burkholderia pseudomallei* and CpG oligodeoxynucleotide against live challenge. Vaccine 32, 5983–5988. 10.1016/j.vaccine.2014.08.035 25223269

[B83] Pukatzki S. Ma A. T. Revel A. T. Sturtevant D. Mekalanos J. J. (2007). Type VI secretion system translocates a phage tail spike-like protein into target cells where it cross-links actin. Proc. Natl. Acad. Sci. U.S.A. 104, 15508–15513. 10.1073/pnas.0706532104 17873062 PMC2000545

[B84] Pumpuang A. Chantratita N. Wikraiphat C. Saiprom N. Day N. P. J. Peacock S.. (2011). Survival of *Burkholderia pseudomallei* in distilled water for 16 years. Trans. R. Soc. Trop. Med. Hyg. 105, 598–600. 10.1016/j.trstmh.2011.06.004 21764093 PMC3183224

[B85] Pumpuang A. Dunachie S. J. Phokrai P. Jenjaroen K. Sintiprungrat K. Boonsilp S.. (2017). Comparison of O-polysaccharide and hemolysin co-regulated protein as target antigens for serodiagnosis of melioidosis. PLoS Negl. Trop. Dis. 11, e0005499. 10.1371/journal.pntd.0005499 28358816 PMC5395236

[B86] Ranasinghe C. Trivedi S. Wijesundara D. K. Jackson R. J. (2014). IL-4 and IL-13 receptors: Roles in immunity and powerful vaccine adjuvants. Cytokine Growth Fact. Rev. 25, 437–442. 10.1016/j.cytogfr.2014.07.010 25159217

[B87] Reynolds C. Goudet A. Jenjaroen K. Sumonwiriya M. Rinchai D. Musson J.. (2015). T cell immunity to the alkyl hydroperoxide reductase of *Burkholderia pseudomallei*: a correlate of disease outcome in acute melioidosis. J. Immunol. 194, 4814–4824. 10.4049/jimmunol.1402862 25862821 PMC4416739

[B88] Rhee J. H. (2020). Current and new approaches for mucosal vaccine delivery. Mucosal. Vaccines 325–356. 10.1016/B978-0-12-811924-2.00019-5 [Epub ahead of print].

[B89] Santanirand P. Harley V. S. Dance D. A. Drasar B. S. Bancroft G. J. (1999). Obligatory role of γ interferon for host survival in a murine model of infection with *Burkholderia pseudomallei*. Infect. Immun. 67, 3593–3600. 10.1128/IAI.67.7.3593-3600.1999 10377144 PMC116549

[B90] Sarkar-Tyson M. Smither S. J. Harding S. V. Atkins T. P. Titball R. W. (2009). Protective efficacy of heat-inactivated *B. thailandensis, B. mallei* or *B. pseudomallei* against experimental melioidosis and glanders. Vaccine 27, 4447–4451. 10.1016/j.vaccine.2009.05.040 19490962

[B91] Sawant K. V. Poluri K. M. Dutta A. K. Sepuru K. M. Troshkina A. Garofalo R. P.. (2016). Chemokine CXCL1 mediated neutrophil recruitment: role of glycosaminoglycan interactions. Sci. Rep. 6, 33123. 10.1038/srep33123 27625115 PMC5021969

[B92] Schmidt L. K. Orne C. E. Shaffer T. L. Wilson S. M. Khakhum N. Torres A. G.. (2022). Development of melioidosis subunit vaccines using and enzymatically inactive *Burkholderia pseudomallei* AhpC. Infect. Immunity 11, e0022222. 10.1128/iai.00222-22 35862715 PMC9387246

[B93] Scott A. E. Burtnick M. N. Stokes M. G. Whelan A. O. Williamson E. D. Atkins T. P.. (2014a). *Burkholderia pseudomallei* capsular polysaccharide conjugates provide protection against acute melioidosis. Infect. Immun. 82, 3206–3213. 10.1128/IAI.01847-14 24866807 PMC4136211

[B94] Scott A. E. Christ W. J. George A. J. Stokes M. G. Lohman G. J. Guo Y.. (2016). Protection against experimental melioidosis with a synthetic manno-heptopyranose hexasaccharide glycoconjugate. Bioconjug. Chem. 27, 1435–1446. 10.1021/acs.bioconjchem.5b00525 27124182 PMC4911622

[B95] Scott A. E. Ngugi S. A. Laws T. R. Corser D. Lonsdale C. L. D'Elia R. V.. (2014b). Protection against experimental melioidosis following immunisation with a lipopolysaccharide-protein conjugate. J. Immunol. Res. 2014, 392170. 10.1155/2014/392170 24892035 PMC4033506

[B96] Sengyee S. Yarasai A. Janon R. Morakot C. Ottiwet O. Schmidt L. K.. (2021). Melioidosis patient survival correlates with strong IFN-γ secreting T cell responses against Hcp1 and TssM. Front. Immunol. 12, 698303. 10.3389/fimmu.2021.698303 34394091 PMC8363298

[B97] Silva E. B. Dow S. W. (2013). Development of *Burkholderia mallei* and pseudomallei vaccines. Front. Cell Infect. Microbiol. 3, 10. 10.3389/fcimb.2013.00010 23508691 PMC3598006

[B98] Silva E. B. Goodyear A. Sutherland M. D. Podnecky N. L. Gonzalez-Juarrero M. Schweizer H. P.. (2013). Correlates of immune protection following cutaneous immunization with an attenuated *Burkholderia pseudomallei* vaccine. Infect. Immun. 81, 4626–4634. 10.1128/IAI.00915-13 24101688 PMC3837973

[B99] Snapper C. M. (2016). Differential regulation of polysaccharide-specific antibody responses to isolated polysaccharides, conjugate vaccines, and intact gram-positive versus gram-negative extracellular bacteria. Vaccine 34, 3542–3548. 10.1016/j.vaccine.2015.12.077 27133879

[B100] Song J. Park D. W. Moon S. Cho H. J. Park J. H. Seok H.. (2019). Diagnostic and prognostic value of interleukin-6, pentraxin 3, and procalcitonin levels among sepsis and septic shock patients: a prospective controlled study according to the Sepsis-3 definitions. BMC Infect. Dis. 19, 968. 10.1186/s12879-019-4618-7 31718563 PMC6852730

[B101] Souza-Fonseca-Guimaraes F. Adib-Conquy M. Cavaillon J. M. (2012). Natural killer (NK) cells in antibacterial innate immunity: angels or devils? Mol. Med. 18, 270–285. 10.2119/molmed.2011.00201 22105606 PMC3324953

[B102] Spellberg B. Edwards J. E. Jr. (2001). Type 1/Type 2 immunity in infectious diseases. Clin. Infect. Dis. 32, 76–102. 10.1086/317537 11118387

[B103] Srilunchang T. Proungvitaya T. Wongratanacheewin S. Strugnell R. Homchampa P. (2009). Construction and characterization of an unmarked *aroC* deletion mutant of *Burkholderia pseudomallei* strain A2. Southeast Asian J. Trop. Med. Public Health 40, 123–130. 19323044

[B104] Srinivasan A. Kraus C. N. DeShazer D. Becker P. M. Dick J. D. Spacek L.. (2001). Glanders in a military research microbiologist. N. Engl. J. Med. 34, 256–258. 10.1056/NEJM200107263450404 11474663

[B105] Stephenson E. H. Moeller R. B. York C. G. Young H. W. (1988). Nose-only versus whole-body aerosol exposure for induction of upper respiratory infections of laboratory mice. Am. Ind. Hyg. Assoc. J. 49, 128–135. 10.1080/15298668891379503 3287878

[B106] Stevens M. P. Wood M. W. Taylor L. A. Monaghan P. Hawes P. Jones P. W.. (2002). An Inv/Mxi-Spa-like type III protein secretion system in *Burkholderia pseudomallei* modulates intracellular behaviour of the pathogen. Mol. Microbiol. 46, 649–659. 10.1046/j.1365-2958.2002.03190.x 12410823

[B107] Tapia D. Sanchez-Villamil J. I. Stevenson H. L. Torres A. G. (2021). Multicomponent gold-linked glycoconjugate vaccine elicits antigen-specific humoral and mixed TH1-TH17 immunity, correlated with increased protection against *Burkholderia pseudomallei*. mBio 12, e0122721. 10.1128/mBio.01227-21 34182777 PMC8263005

[B108] Testamenti V. A. Noviana R. Iskandriati D. Norris M. H. Jiranantasak T. Tuanyok A.. (2020). Humoral immune responses to *Burkholderia pseudomallei* antigens in captive and wild macaques in the western part of Java, Indonesia. Vet. Sci. 7, 153. 10.3390/vetsci7040153 33050516 PMC7712568

[B109] Tippayawat P. Saenwongsa W. Mahawantung J. Suwannasaen D. Chetchotisakd P. Limmathurotsakul D.. (2009). Phenotypic and functional characterization of human memory T cell responses to *Burkholderia pseudomallei*. PLoS Negl. Trop. Dis. 3, e407. 10.1371/journal.pntd.0000407 19352426 PMC2660609

[B110] Tisoncik J. R. Korth M. J. Simmons C. P. Farrar J. Martin T. R. Katze M. G.. (2012). Into the eye of the cytokine storm. Microbiol. Mol. Biol. Rev. 76, 16–32. 10.1128/MMBR.05015-11 22390970 PMC3294426

[B111] Titball R. W. Burtnick M. N. Bancroft G. J. Brett P. (2017). *Burkholderia pseudomallei* and *Burkholderia mallei* vaccines: are we close to clinical trials? Vaccine 35, 5981–5989. 10.1016/j.vaccine.2017.03.022 28336210

[B112] Tomas-Cortazar J. Bossi L. Quinn C. Reynolds C. J. Butler D. K. Corcoran N.. (2021). BpOmpW antigen stimulates the necessary protective T-cell responses against melioidosis. Front. Immunol. 12, 767359. 10.3389/fimmu.2021.767359 34966388 PMC8710444

[B113] Trevino S. R. Dankmeyer J. L. Fetterer D. P. Klimko C. P. Raymond J. L. W. Moreau A. M.. (2021). Comparative virulence of three different strains of *Burkholderia pseudomallei* in an aerosol non-human primate model. PLoS Negl. Trop. Dis. 15, e0009125. 10.1371/journal.pntd.0009125 33571211 PMC7904162

[B114] Trevino S. R. Klimko C. P. Reed M. C. Aponte-Cuadrado M. J. Hunter M. Shoe J. L.. (2018). Disease progression in mice exposed to low-doses of aerosolized clinical isolates of *Burkholderia pseudomallei*. PLoS ONE 13, e0208277. 10.1371/journal.pone.0208277 30500862 PMC6267979

[B115] Valeri M. Raffatellu M. (2016). Cytokines IL-17 and IL-22 in the host response to infection. Pathog Dis. 74, ftw111. 10.1093/femspd/ftw111 27915228 PMC5975231

[B116] Van Zandt K. E. Tuanyok A. Keim P. S. Warren R. L. Gelhaus H. C. (2012). An objective approach for *Burkholderia pseudomallei* strain selection as challenge material for medical countermeasures efficacy testing. Front. Cell Infect. Microbiol. 2, 120. 10.3389/fcimb.2012.00120 23057010 PMC3458228

[B117] Varma T. K. Lin C. Y. Toliver-Kinsky T. E. Sherwood E. R. (2002). Endotoxin-induced γ interferon production: contributing cell types and key regulatory factors. Clin. Diagn. Lab Immunol. 9, 530–543. 10.1128/CDLI.9.3.530-543.2002 11986256 PMC119981

[B118] Wang G. Zarodkiewicz P. Valvano M. A. (2020). Current advances in Burkholderia vaccines development. Cells 9, 2671. 10.3390/cells9122671 33322641 PMC7762980

[B119] Warawa J. M. Long D. Rosenke R. Gardner D. Gherardini F. C. (2009). Role for the *Burkholderia pseudomallei* capsular polysaccharide encoded by the wcb operon in acute disseminated melioidosis. Infect. Immun. 77, 5252–5261. 10.1128/IAI.00824-09 19752033 PMC2786491

[B120] Welkos S. L. Klimko C. P. Kern S. J. Bearss J. J. Bozue J. A. Bernhards R. C.. (2015). Characterization of *Burkholderia pseudomallei* strains using a murine intraperitoneal infection model and *in vitro* macrophage assays. PLoS ONE 10, e0124667. 10.1371/journal.pone.0124667 25909629 PMC4409376

[B121] Whitlock G. C. Lukaszewski R. A. Judy B. M. Paessler S. Torres A. G. Estes D. M.. (2008). Host immunity in the protective response to vaccination with heat-killed *Burkholderia mallei*. BMC Immunol. 9, 55. 10.1186/1471-2172-9-55 18823549 PMC2562362

[B122] Wiersinga W. J. Currie B. J. Peacock S. J. (2012). Melioidosis. N. Engl. J. Med. 367, 1035–1044. 10.1056/NEJMra1204699 22970946

[B123] Wijesundara D. K. Jackson R. J. Tscharke D. C. Ranasinghe C. (2013). IL-4 and IL-13 mediated down-regulation of CD8 expression levels can dampen anti-viral CD8(+) T cell avidity following HIV-1 recombinant pox viral vaccination. Vaccine 31, 4548–4555. 10.1016/j.vaccine.2013.07.062 23933364

[B124] Wright S. W. Kaewarpai T. Lovelace-Macon L. Ducken D. Hantrakun V. Rudd K. E.. (2021). A 2-biomarker model augments clinical prediction of mortality in melioidosis. Clin. Infect. Dis. 72, 821–828. 10.1093/cid/ciaa126 32034914 PMC7935382

[B125] Yeh H. C. Snipes M. B. Eidson A. F. Hobbs C. H. Henry M. C. (1990). Comparative evaluation of nose-only versus whole-body inhalation exposures for rats—aerosol characteristics and lung deposition, inhalation *Toxicology* 2, 205–221. 10.3109/08958379009145255

[B126] Yi J. Herring K. Sanchez T. C. Iyer S. Stone J. K. Lee J.. (2016). Immunological patterns from four melioidosis cases: constant and variable protein antigens. bioRxiv 2016, 20161120. 10.1101/082057

