# Supplementary material for: Evaluation of two different vaccine platforms for immunization against melioidosis and glanders
Source: Front Microbiol. 2022 Aug 17;13:965518. doi: 10.3389/fmicb.2022.965518 (PMC9428723; doi:10.3389/fmicb.2022.965518)
Supplement: Supplementary file 1 [file Presentation_1.pptx]

## Slide 1
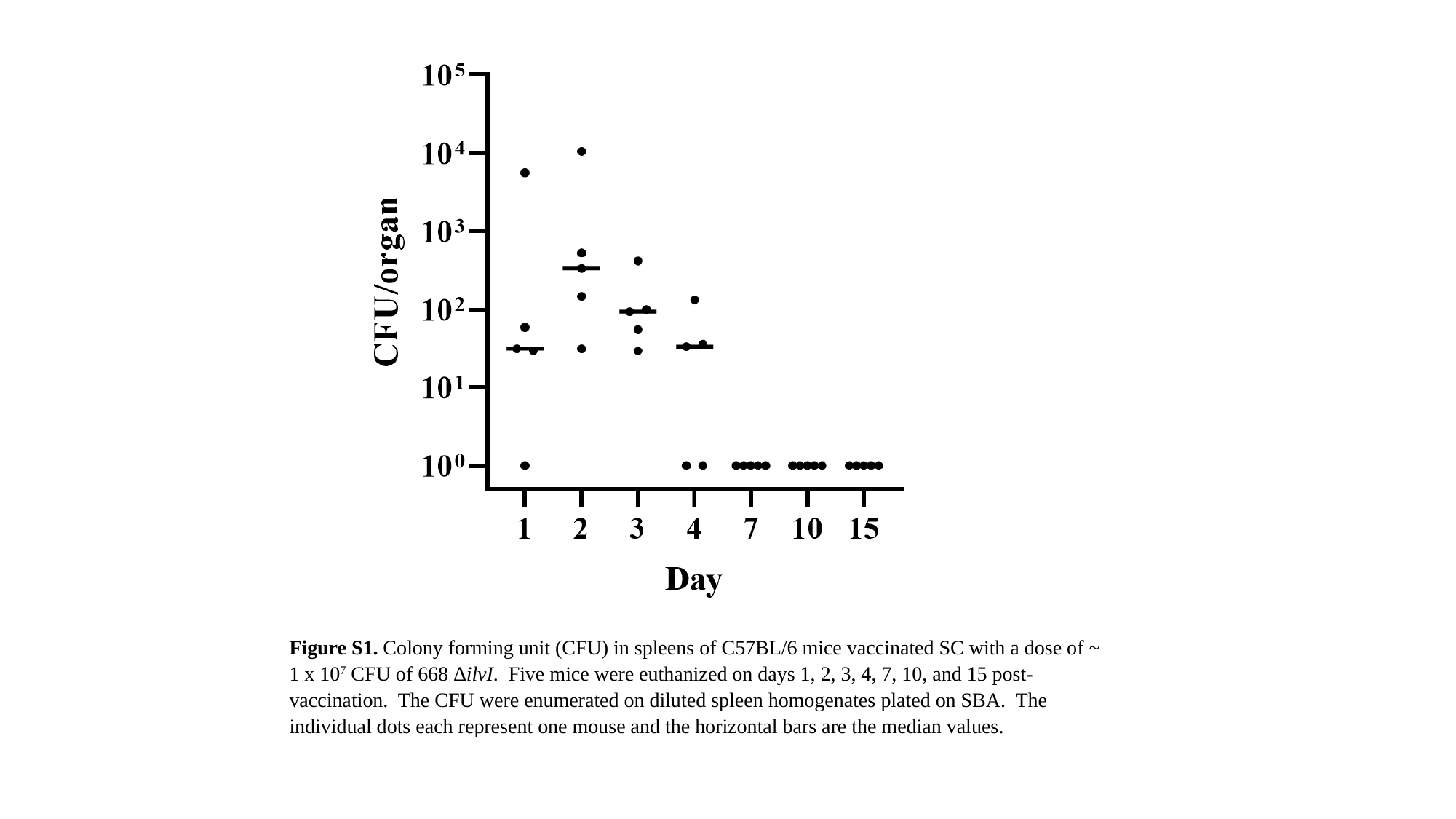

Figure S1. Colony forming unit (CFU) in spleens of C57BL/6 mice vaccinated SC with a dose of ~ 1 x 10^7^ CFU of 668 ∆ilvI. Five mice were euthanized on days 1, 2, 3, 4, 7, 10, and 15 post-vaccination. The CFU were enumerated on diluted spleen homogenates plated on SBA. The individual dots each represent one mouse and the horizontal bars are the median values.

## Slide 2
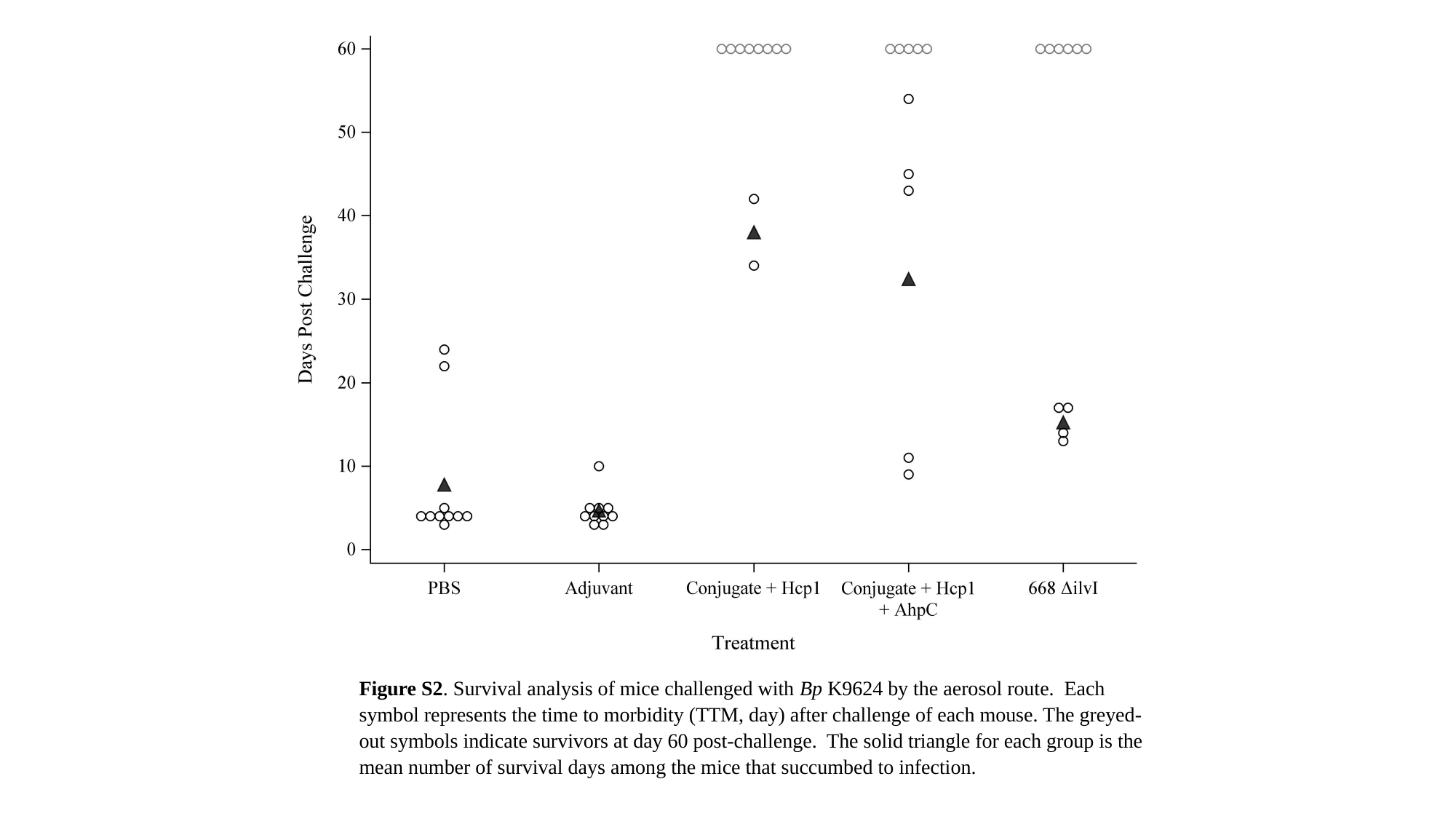

Figure S2. Survival analysis of mice challenged with Bp K9624 by the aerosol route. Each symbol represents the time to morbidity (TTM, day) after challenge of each mouse. The greyed-out symbols indicate survivors at day 60 post-challenge. The solid triangle for each group is the mean number of survival days among the mice that succumbed to infection.

## Slide 3
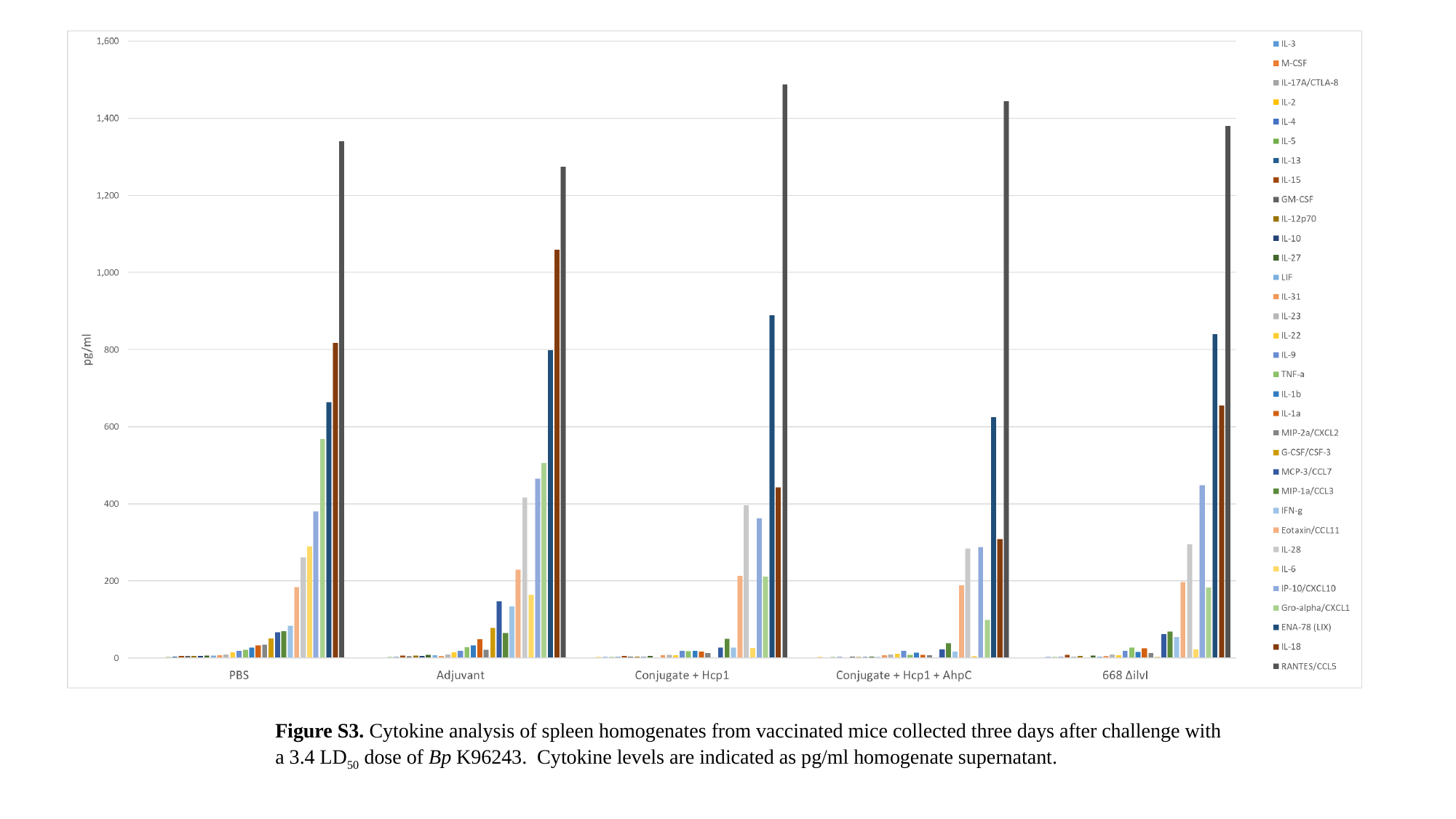

Figure S3. Cytokine analysis of spleen homogenates from vaccinated mice collected three days after challenge with a 3.4 LD50 dose of Bp K96243. Cytokine levels are indicated as pg/ml homogenate supernatant.

## Slide 4
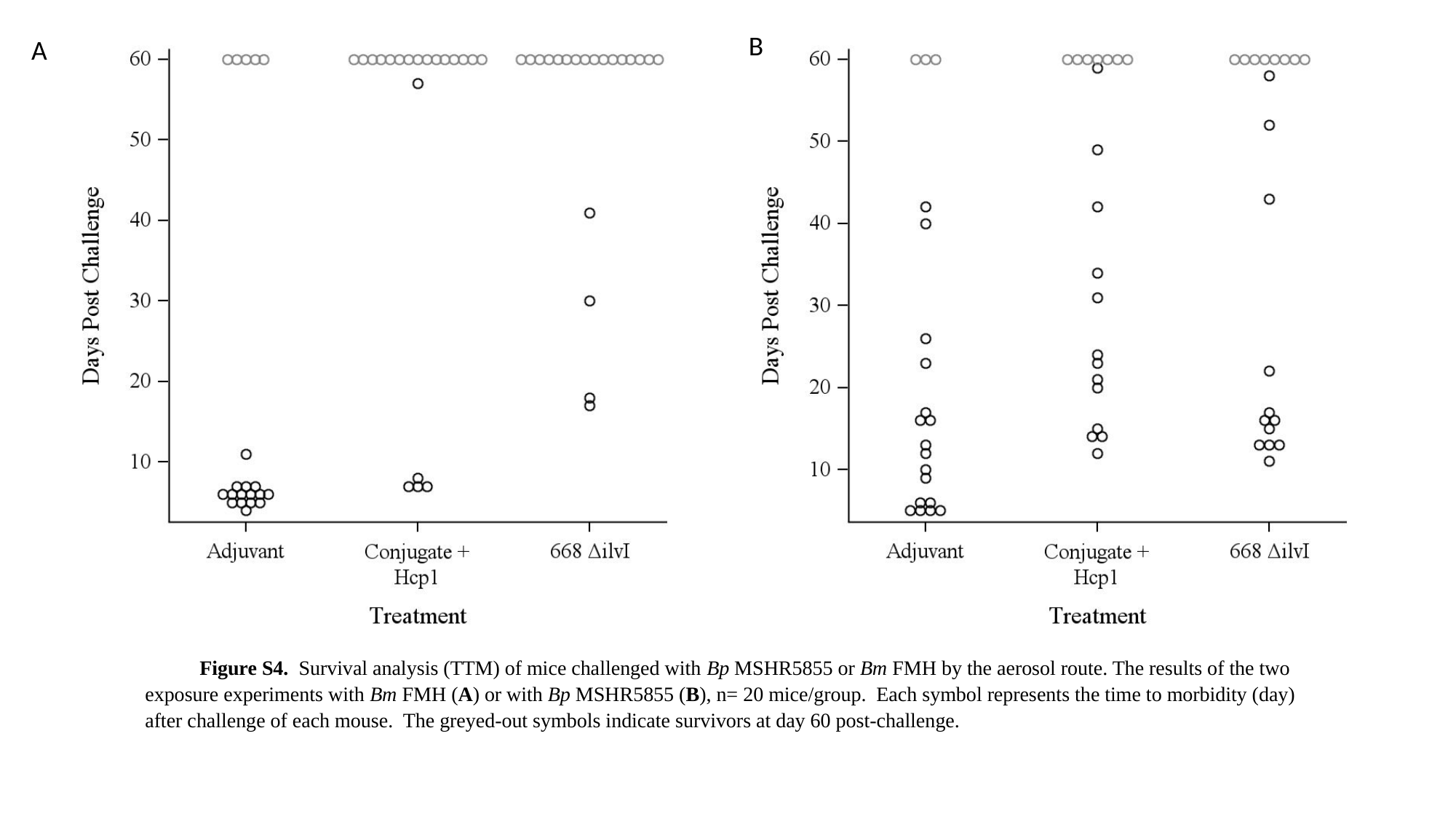

B
A
Figure S4. Survival analysis (TTM) of mice challenged with Bp MSHR5855 or Bm FMH by the aerosol route. The results of the two exposure experiments with Bm FMH (A) or with Bp MSHR5855 (B), n= 20 mice/group. Each symbol represents the time to morbidity (day) after challenge of each mouse. The greyed-out symbols indicate survivors at day 60 post-challenge.

## Slide 5
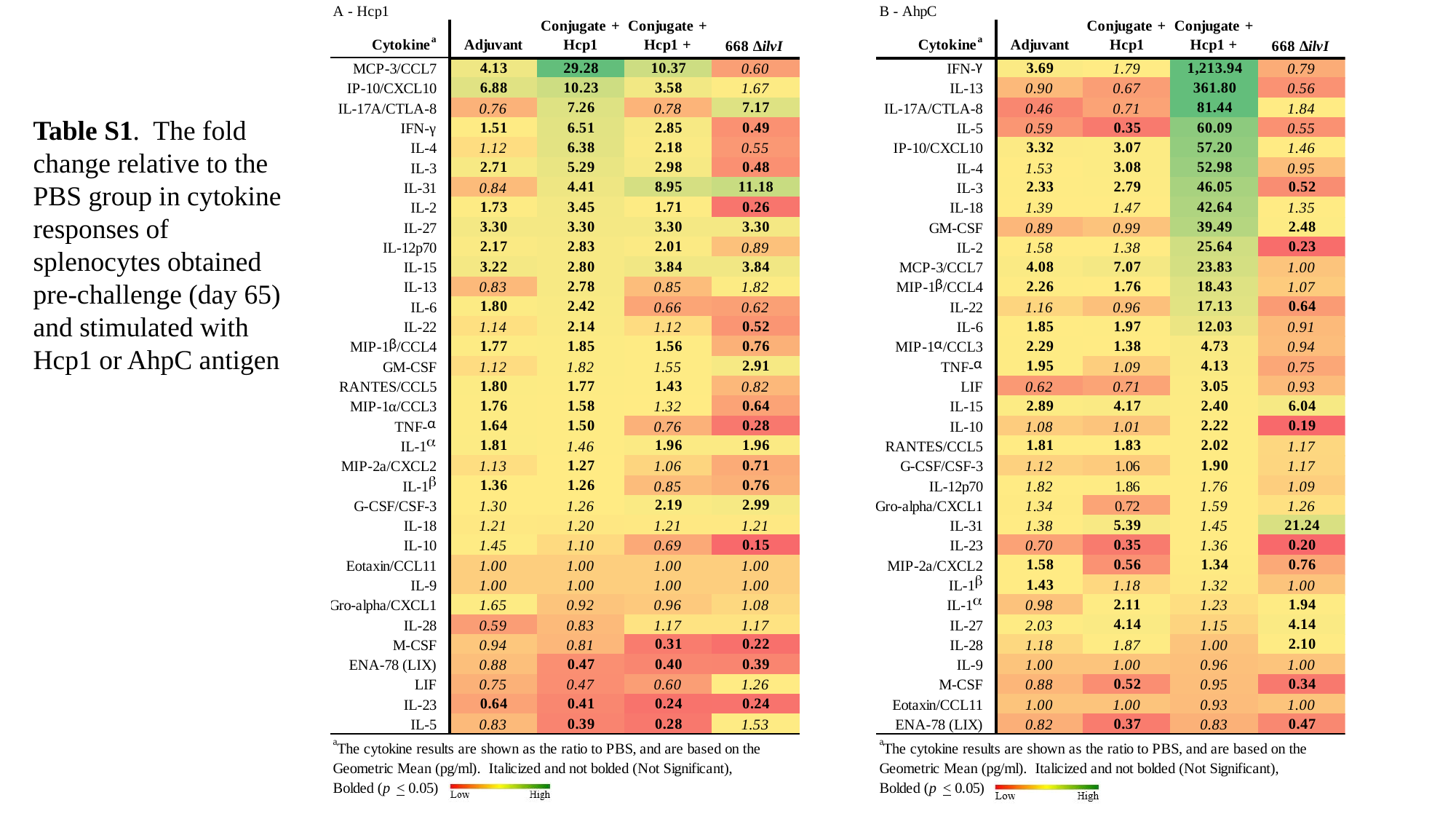

Table S1. The fold change relative to the PBS group in cytokine responses of splenocytes obtained pre-challenge (day 65) and stimulated with Hcp1 or AhpC antigen

## Slide 6
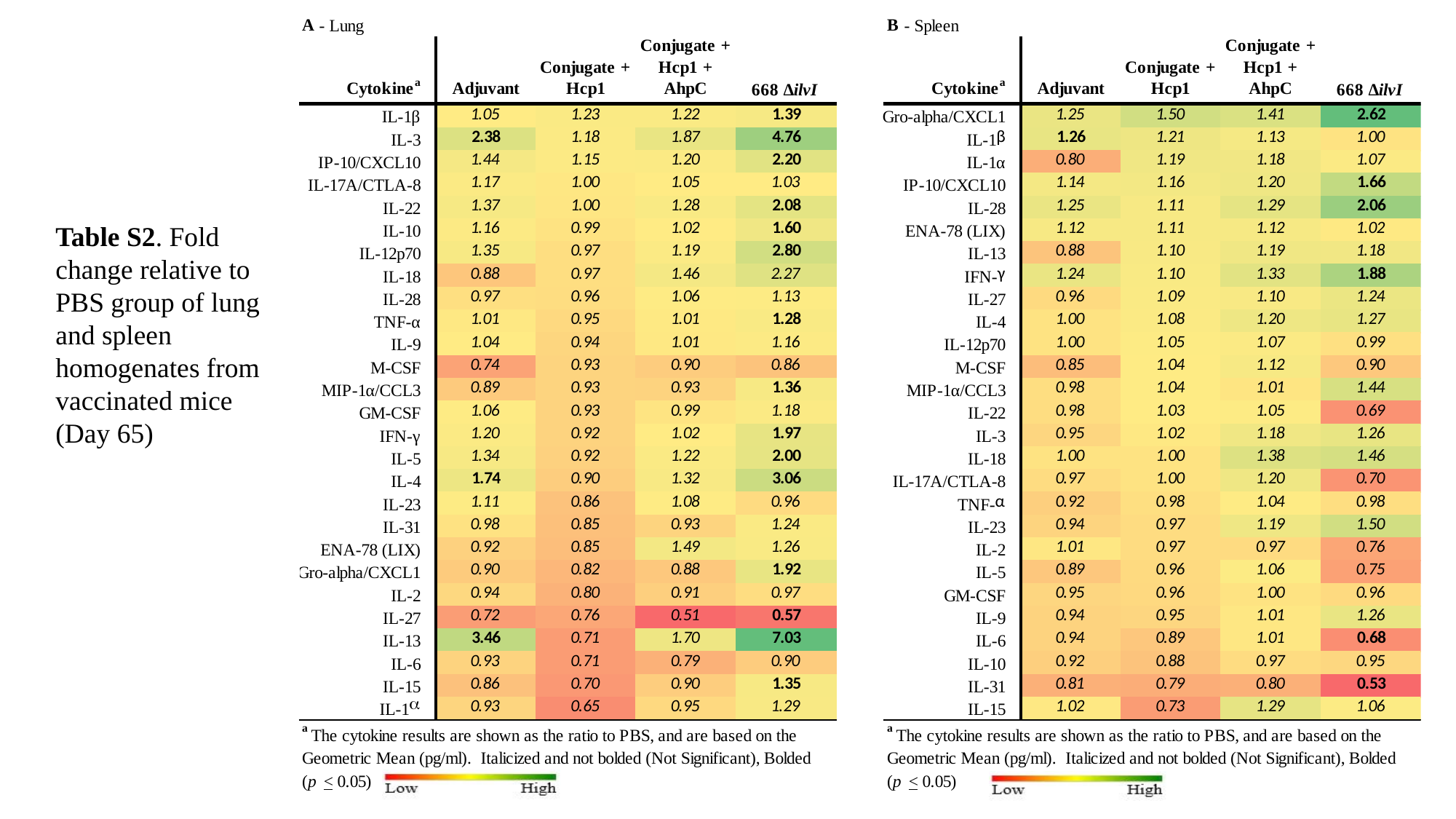

Table S2. Fold change relative to PBS group of lung and spleen homogenates from vaccinated mice (Day 65)

## Slide 7
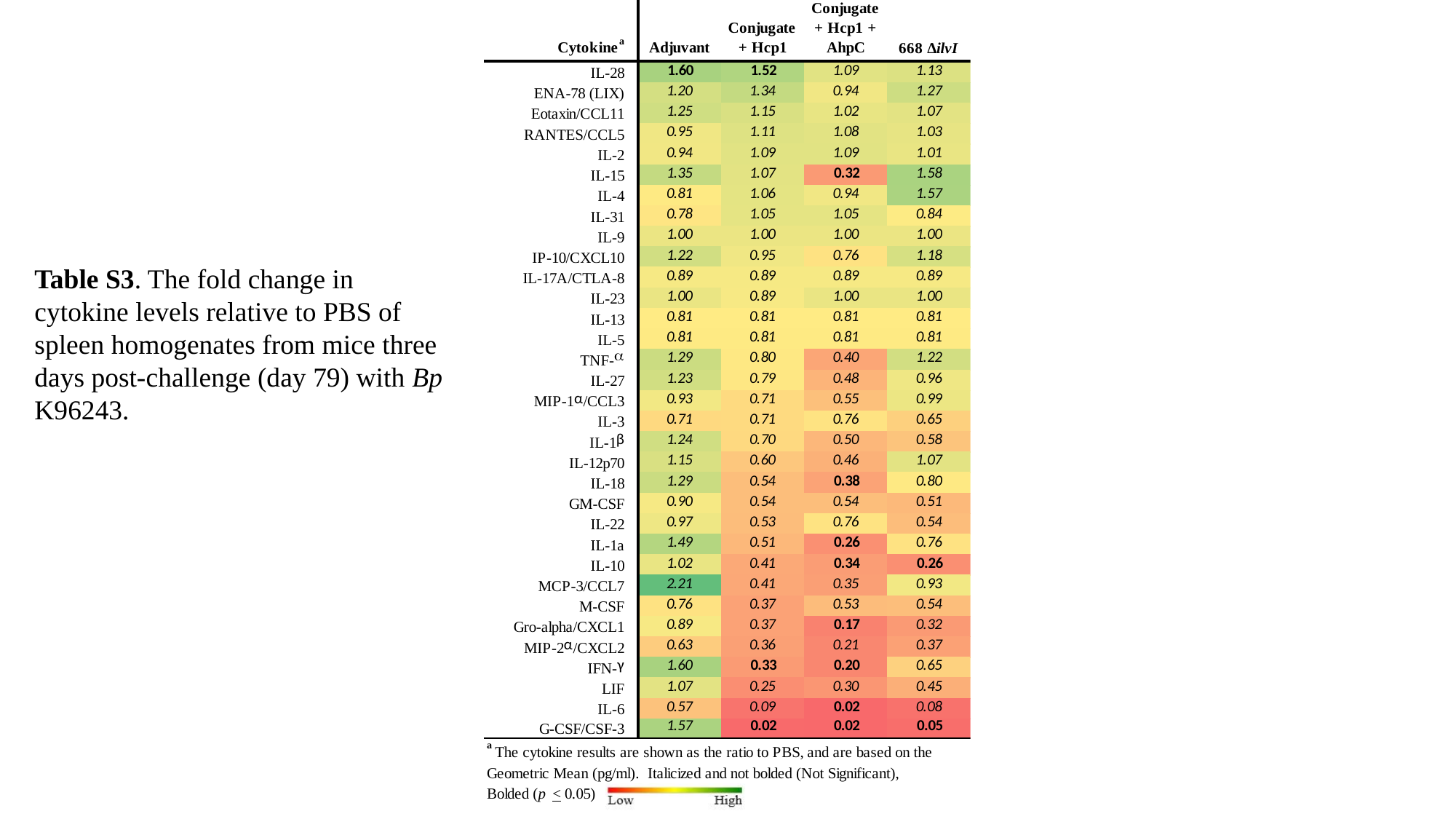

Table S3. The fold change in cytokine levels relative to PBS of spleen homogenates from mice three days post-challenge (day 79) with Bp K96243.

## Slide 8
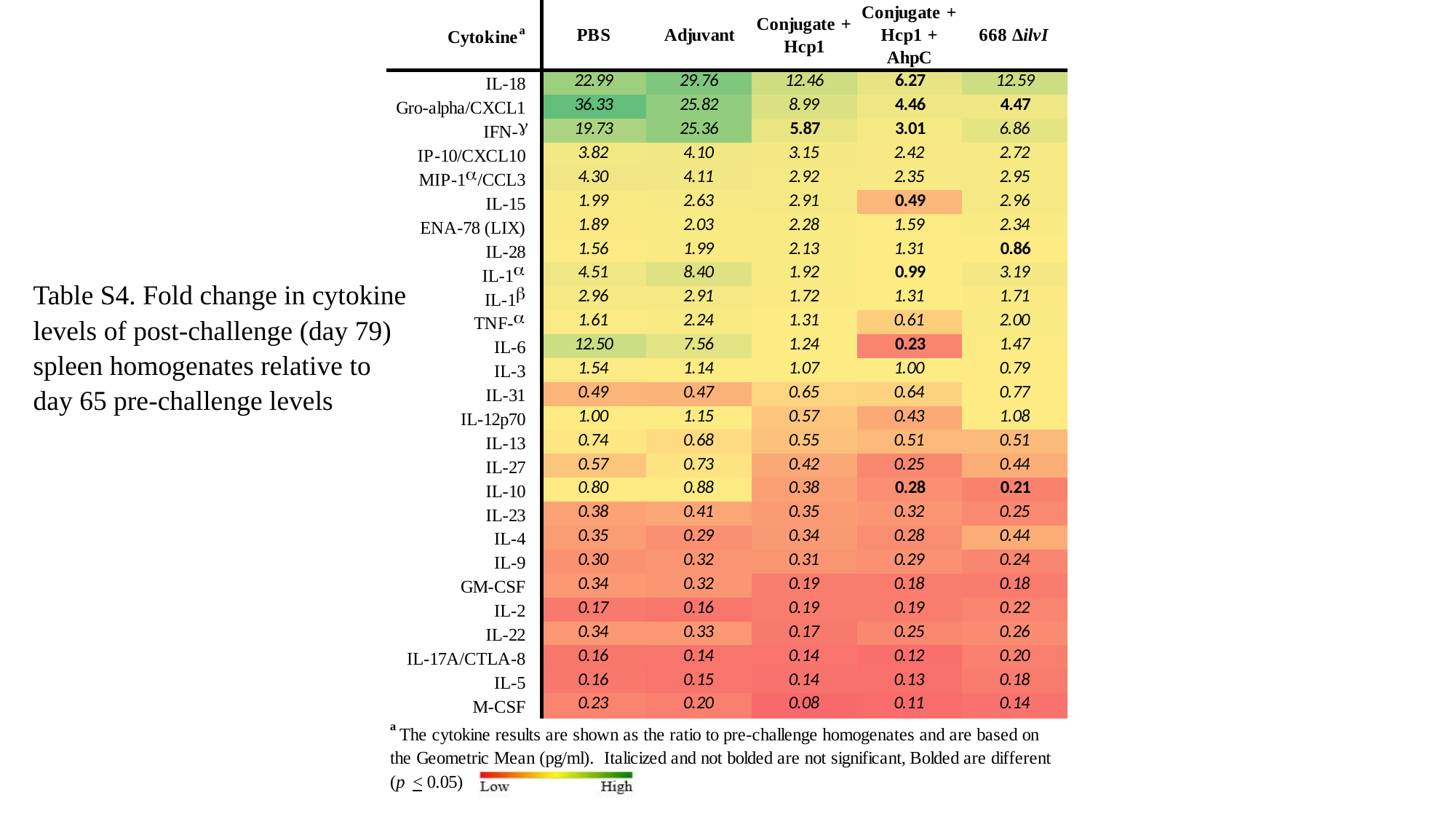

Table S4. Fold change in cytokine levels of post-challenge (day 79) spleen homogenates relative to day 65 pre-challenge levels

## Slide 9
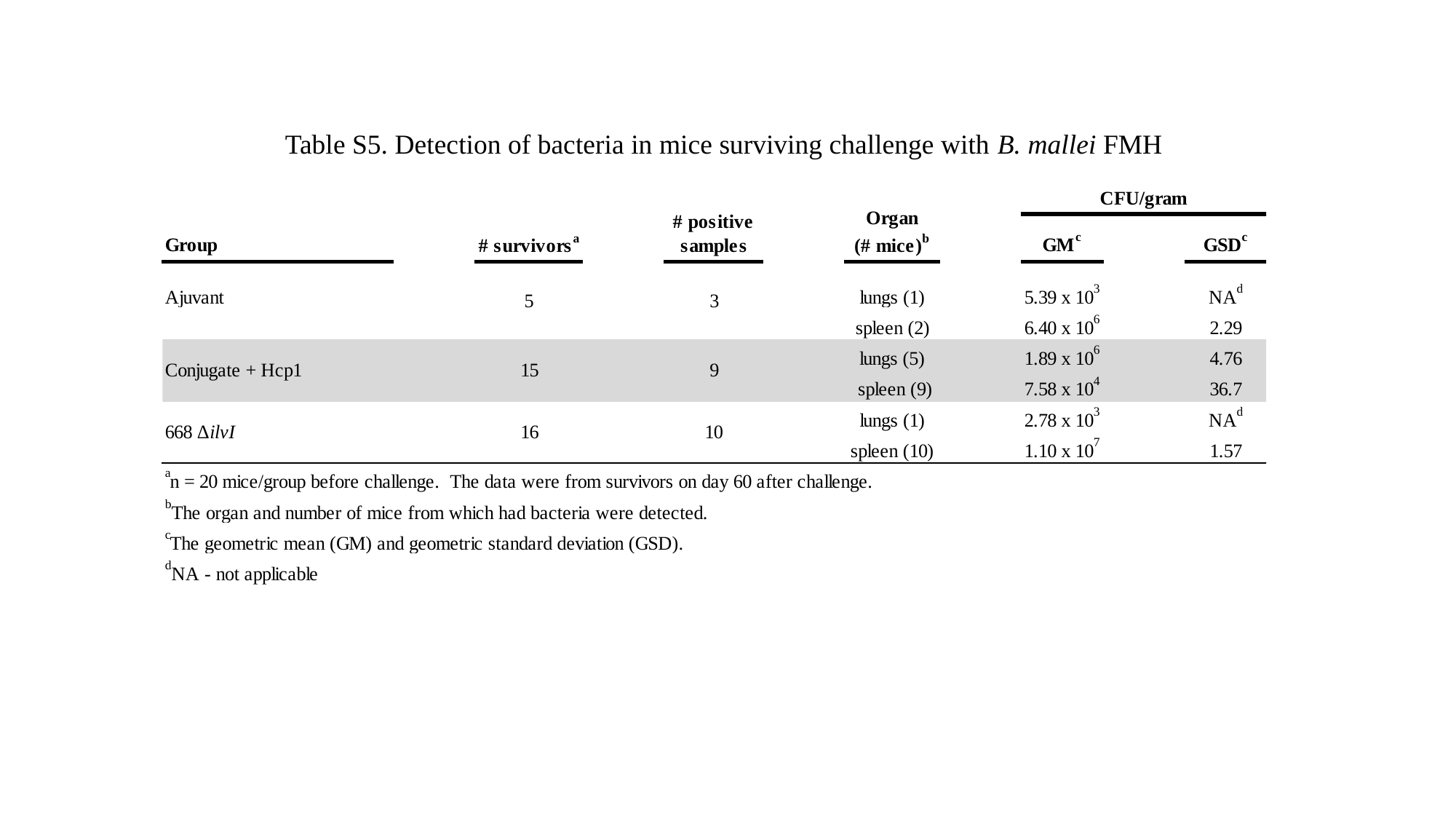

Table S5. Detection of bacteria in mice surviving challenge with B. mallei FMH

## Slide 10
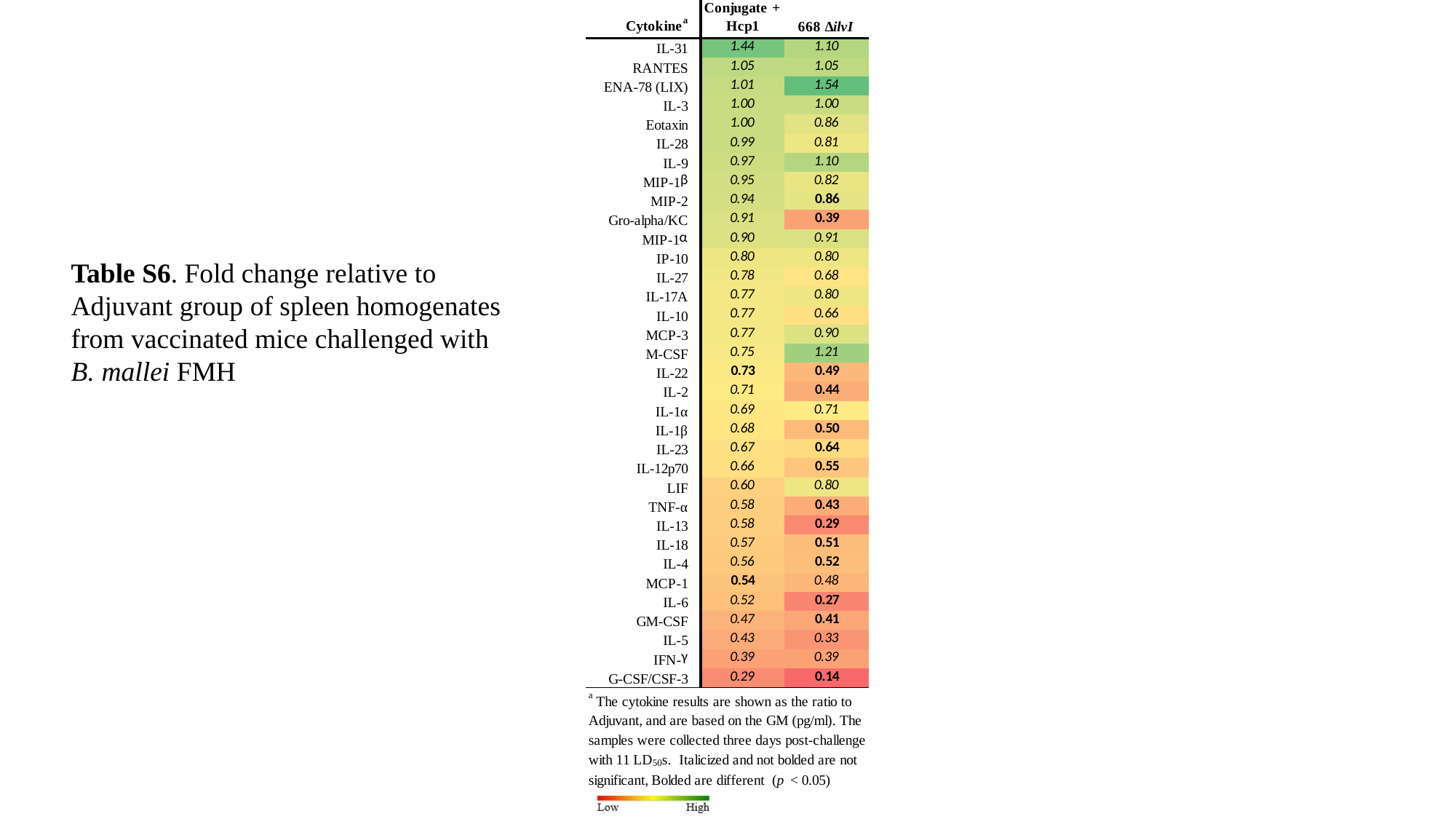

Table S6. Fold change relative to Adjuvant group of spleen homogenates from vaccinated mice challenged with B. mallei FMH

## Slide 11
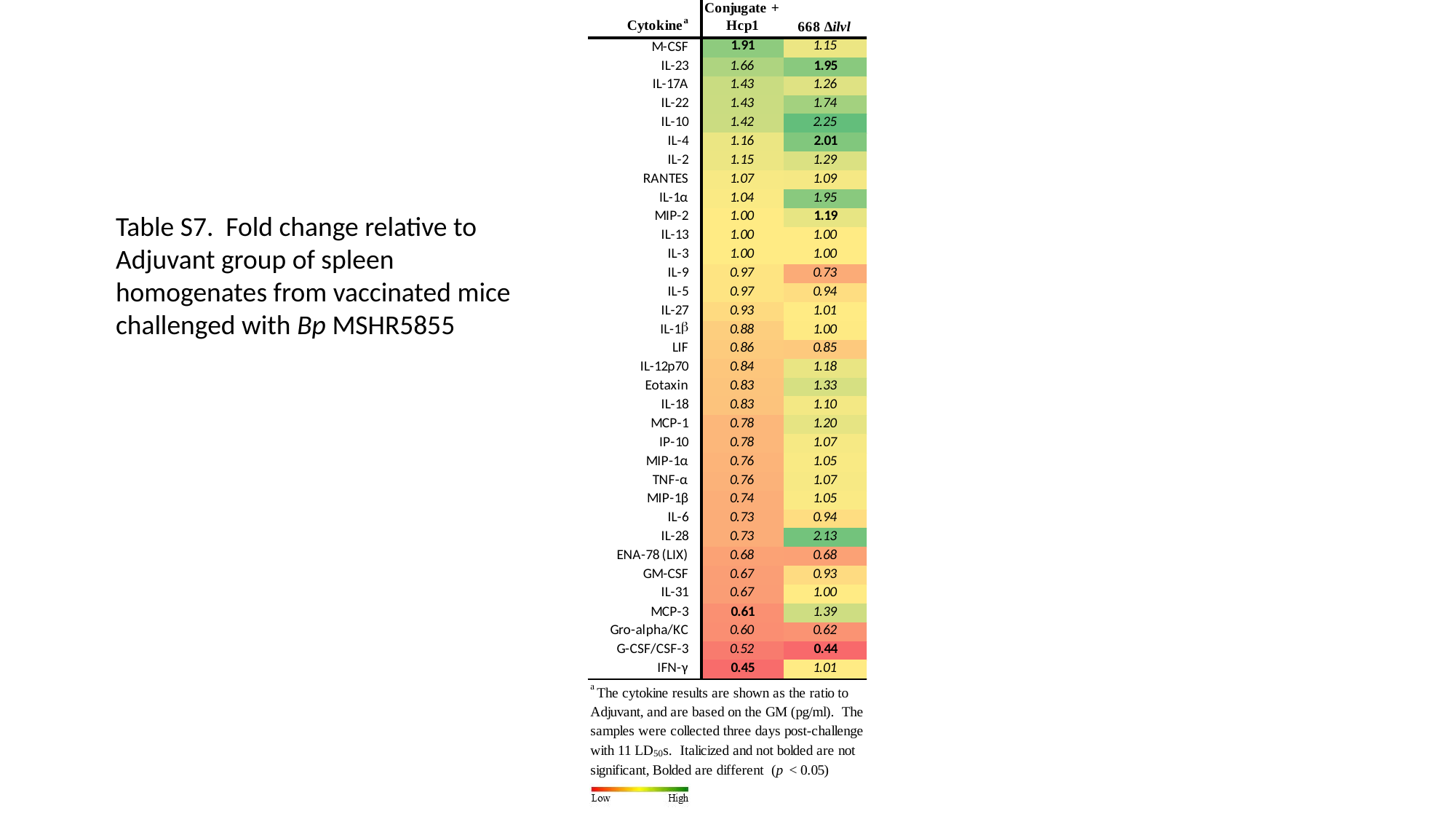

Table S7. Fold change relative to Adjuvant group of spleen homogenates from vaccinated mice challenged with Bp MSHR5855
